# TNAP and PHOSPHO1 Function Synergistically to Afford Critical Control Over the Mineralization of the Postnatal Murine Skeleton

**DOI:** 10.1002/advs.77671

**Published:** 2026-09-09

**Authors:** Lucie E. Bourne, Aikta Sharma, Scott Dillon, Jacob Keen, Soher N. Jayash, Natalie Crump, Lucinda A.E. Evans, Maya Karmali, Worachet Promruk, Claire E. Clarkin, Sonoko Narisawa, Louise Stephen, Brian L. Foster, José Luis Millán, Colin Farquharson, Katherine A. Staines

**Affiliations:** ^1^ Centre For Lifelong Health School of Applied Sciences University of Brighton Brighton UK; ^2^ Department of Mechanical Engineering University College London London UK; ^3^ Yusuf Hamied Department of Chemistry University of Cambridge Cambridge UK; ^4^ School of Biological Sciences University of Southampton Southampton UK; ^5^ The Roslin Institute and Royal (Dick) School of Veterinary Studies University of Edinburgh Midlothian UK; ^6^ Faculty of Life Sciences & Medicine Kings College London London UK; ^7^ Human Genetics Program Sanford Burnham Prebys Medical Discovery Institute La Jolla California USA; ^8^ Division of Biosciences College of Dentistry The Ohio State University Columbus Ohio USA

**Keywords:** biomineralization, bone, murine model, Phospho1, Tissue non‐specific alkaline phosphatase

## Abstract

Biomineralization is essential for skeletal integrity, yet the synergistic roles of tissue non‐specific alkaline phosphatase (TNAP) and PHOSPHO1 in postnatal bone remain unclear. We generated a novel murine model with *Prx1*‐driven deletion of *Alpl* (*Alpl^Prx1/Prx1^*) on a global *Phospho1* ^−/−^ background, circumventing the perinatal lethality associated with dual global deletion and premature death of *Alpl* global knockouts. Multi‐modal spatial phenotyping of the limbs revealed that mice lacking both TNAP and PHOSPHO1 exhibit distinct mineralization defects and altered anatomical structures at postnatal day 1 (PN1) and 3‐weeks of age. Although viable, these mice did not thrive due to reduced size; thus further investigations were conducted on mice with heterozygous deletion of TNAP (*Alpl^wt/Prx1^;Phospho1^−/−^*). Although smaller than wild‐types at PN1 and 3 weeks old, these mice preserved gross limb structure and the single, functioning *Alpl* allele rescued biomineralization loss following dual phosphatase deletion. In 6‐week animals, compromised epiphyses and metaphyses were only observed in *Alpl^Prx1/Prx1^* animals. Conversely, diaphyseal geometry and porosity were altered by *Phospho1* deletion, compounded in *Alpl^wt/Prx1^;Phospho1^−/‒^* mice, and linked to alterations in collagen configuration, matrix mineralization, and growth plate deformities. Together, our findings support a mechanistic framework for TNAP and PHOSPHO1 in permissive biomineralization, providing critical insights into this fundamental process.

## Graphical abstract

Biomineralization underpins skeletal development, yet its molecular control remains incompletely understood. Using a novel murine knockout model, this study reveals the essential and complementary roles of PHOSPHO1 and TNAP in postnatal skeletal development. Distinct spatial functions within long bones uncover new biological insights and highlight potential therapeutic avenues for disorders of abnormal bone mineralization.

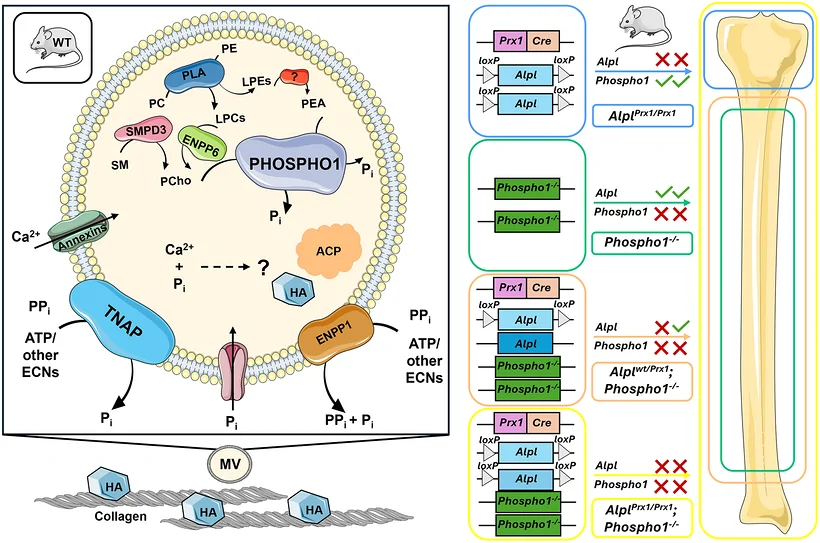

## 1 Introduction

Biomineralization of the skeleton is indispensable for vertebrate health and wellbeing throughout life. This involves a series of tightly regulated physicochemical and biochemical processes that facilitate the deposition of carbonated hydroxyapatite mineral crystals within the organic collagenous matrix of bone [1, 2]. Local promoters of mineralization are synthesized by osteoblasts and comprise of several phosphatases, with seminal studies showing tissue non‐specific alkaline phosphatase (TNAP) and phosphoethanolamine/phosphocholine phosphatase 1 (PHOSPHO1) being central to this process [3, 4, 5, 6, 7]. Although converging mechanisms between these phosphatases have been proposed [8, 9, 10, 11], how these interact and function to control the initiation and regulation of biomineralization remains incomplete, representing a critical gap in our understanding of *de novo* bone formation.

Characterized by a hypomineralized bone matrix, missense mutations in the human TNAP (*ALPL*) gene lead to hypophosphatasia (HPP), a heritable form of rickets, and osteomalacia [7]. Similarly, global deletion of *Alpl* in mice (*Alpl* ^−/−^) phenocopies infantile hypophosphatasia. *Alpl* ^−/−^ mice exhibit normal skeletal mineralization at birth, yet by postnatal day 6, hypomineralization becomes apparent and worsens until their early demise by postnatal day 20 [12, 13]. This aberrant mineralization is, in part, driven by an accumulation of extracellular inorganic pyrophosphate (ePP_i_), a physiological substrate of TNAP and potent mineralization inhibitor, which arrests mineral crystal propagation onto the collagen‐rich extracellular matrix [14, 15]. ePP_i_ is produced ectoplasmically via cellular efflux of ATP from Ankylosis protein (ANK) and by the enzymatic action of ectonucleotide pyrophosphatase/phosphodiesterase‐1 (ENPP1) that catabolizes extracellular ATP into ePP_i_ and AMP [16, 17, 18]. TNAP generates P_i_ from ePP_i_ while simultaneously restricting the concentration of ePP_i_ to maintain the PP_i_/P_i_ ratio permissive for bone mineralization [16]. TNAP can also promote matrix mineralization by dephosphorylating the phosphorylated form of osteopontin (OPN), another potent mineralization inhibitor [19, 20]. To phenocopy late‐onset HPP in skeletally mature animals, conditional deletion of *Alpl* using *Cre* recombinases in *Col1a1‐* (osteoblasts and dental cells), *Prx1‐* (early limb‐bud and craniofacial mesenchyme) and *P0* (cranial neural crest) expressing cells has revealed long bone and skull shape defects, hypomineralization and dentoalveolar disease [21, 22]. Small membrane bound matrix vesicles (MVs) provide an ideal environment for the formation of calcium‐phosphate complexes, however, osteoblast‐derived MVs in both hypophosphatasia patients and *Alpl* ^−/−^ mice retain the ability to initiate intra‐vesicular mineral formation, demonstrating that TNAP is not essential for the initiation of mineralization [11, 23, 24].

PHOSPHO1 is the only known phosphatase specific to bone and cartilage [5, 25, 26, 27, 28, 29]. As a member of the haloacid dehalogenase superfamily of enzymes that functions as an intra‐vesicular phosphatase, PHOSPHO1 exhibits high specificity toward phosphoethanolamine and phosphocholine (PCho) which are enriched in MV membranes [30, 31, 32, 33, 34]. These PHOSPHO1 substrates enable the release of P_i_ inside MVs to promote mineral formation [31, 35]. Genetic ablation of *Phospho1* in mice results in elevated ePP_i_ levels, osteomalacia, spontaneous fractures, spinal deformities and bowed long bone [11, 36]. In contrast to the skeletal phenotype of *Alpl* or *Phospho1*‐deficient mice, the simultaneous global genetic ablation of both phosphatases results in an entirely unmineralized skeleton and perinatal death [11]. This observation highlights that while TNAP and PHOSPHO1 function independently during matrix mineralization, further interrogation of their roles will yield mechanistic insights into the initiation of skeletal mineralization [9, 11, 37].

Since mice lacking both *Alpl* and *Phospho1* die perinatally, it is unclear whether these phosphatases synergize to regulate bone mineralization and skeletal structure in the postnatal skeleton. Mineralization of the collagen matrix in the embryonic and postnatal skeleton differs in several key aspects and therefore need to be considered independently [38]. Moreover, the growing postnatal skeleton is subject to extensive modelling events which are reliant on the mineralization of newly formed osteoid. To fully comprehend the functional role of TNAP and PHOSPHO1 in the mineralization of the postnatal skeleton, we circumvented the perinatal lethality of the *Alpl* ^−/−^;*Phospho1* ^−/−^ global knockout by generating mice with a conditional osteoblast‐specific deletion of *Alpl* on a global *Phospho1* ^−/−^ genetic background (*Alpl^Prx1/Prx1^;Phospho1^−/−^*).

## 2 Materials and Methods

### 2.1 Reagents

Unless otherwise stated, all reagents were obtained from ThermoFisher Scientific, and chemicals were purchased from Merck.

### 2.2 Animals

All tissue isolation and experimental procedures were performed in accordance with the UK Animals (Scientific Procedures) Act of 1986 and regulations set by the UK Home Office and local institutional guidelines at the University of Brighton.

Tissue‐specific *Alpl^Prx1/Prx1^* mice were generated using the *Prx1‐Cre* transgene as previously reported [21]. Similarly, the generation and characterization of Phospho1‐R74X mutant mice (Phospho1^m1Jlm^, here referred to as *Phospho1^−/−^)* were also previously described [11]. To generate mice lacking global PHOSPHO1 and *Prx1*‐conditional TNAP, *Phospho1^−/− ^* and *Alpl^Prx1/Prx1^* were crossed to generate double mutant (*Alpl^Prx1/Prx1^*;*Phospho1^−/−^*) and *Alpl*‐heterozygous (*Alpl^wt/Prx1^*;*Phospho1^−/−^*) mice. Genotyping was conducted by Transnetyx using their automated genotyping service. Tissues were collected from both male and female mice at postnatal day 1 (PN1), 3‐ and 6‐ weeks of age, following euthanasia by Schedule 1. Analyses were conducted blindly to minimize the effects of subjective bias.

### 2.3 Whole Mount Staining

PN1 and 3‐week‐old animals were skinned, eviscerated, and fixed in 95% ethanol overnight, as previously described [39]. Acetone (100%) was replaced for 1–2 days. Preparations were incubated in 0.03% alcian blue (w/v), 80% ethanol, 20% glacial acetic acid for 1–3 days, before destaining in two changes of 70% ethanol and incubating in 95% ethanol overnight. Samples were pre‐cleared in 1% potassium hydroxide (KOH) solution overnight at 4°C and incubated in 0.005% alizarin red (in 1% KOH) at 4°C for 1–3 days. Specimens were cleared in 1% KOH and stored in 50% glycerol:50% KOH (1%) before imaging.

### 2.4 MicroCT (µCT) Analysis

Whole body scans of PN1 (n = 3/genotype) and 3‐week‐old animals (n = 3/genotype), and 6‐week‐old left tibiae (n = 5‐7/genotype/sex) were performed using a Skyscan 1172 X‐Ray microtomograph (Bruker). Tomographic scans (200 µA, 0.5 mm aluminium filter, 0.6° rotation angle) were acquired with a voxel size of 10 µm (40 kV) for PN1 animals; 13.5 µm (50 kV) for 3‐week‐old animals; 5 µm for 6‐week‐old tibiae (40 kV). The projection images were reconstructed using NRecon software (Bruker, version 1.7.4.6). Each dataset was orientated and aligned in DataViewer (Bruker, version 1.5.6.2). Region of interest segmentation was performed in CTAn software (Skyscan).

In PN1 and 3‐week‐old mice, bone surface area of the entire skeleton was performed on binarized images using a minimum threshold of 80. 3D measurements were obtained in CTAn. In tibiae, 3D analysis of the trabecular bone was performed using a volume of interest of 5% of the total bone length, as previously described [40]. This region extended distally from the bottom of the growth plate where the primary spongiosa is no longer visible, toward the diaphysis. Analysis of the epiphysis included the most proximal part of the tibia to the unmineralized growth plate. A binarized image with a minimum threshold set to 85 was used across all datasets, and 3D measurements taken in CTAn. BMD measurements were calibrated with appropriate phantoms. Cortical bone was analyzed using a 2D whole‐bone approach to visualize changes across the entire tibia [41]. To ensure exclusion of the trabecular bone, the proximal 15% and distal 10% of the tibial length were excluded. Slice‐by‐slice measurements were created in CTAn using binarized images with a minimum threshold set to 80. Volumetric tissue mineral density (TMD) measurements were calibrated using appropriate phantoms. 3D‐reconstructed images for the entire skeleton, and the cortical and trabecular regions of the tibia were created using Avizo software (ThermoFisher Scientific).

Growth plate bridge analysis was conducted as previously described, whereby the central points of all bony bridges were identified and projected on the tibial joint surface to determine spatial distribution [41]. To evaluate 3D growth plate volume (µm^3^) and thickness (µm, parallel plate model), proximal tibial growth plates were manually segmented using Avizo. To generate false‐color thickness heatmaps, segmentations were resampled, and the Avizo ‘thickness map’ module with the ‘physics’ Lookup Table applied.

To further examine cortical porosity, tibial µCT scans (500 slices from the tibiofibular junction) were processed in FIJI ImageJ. Thresholding produced binary images, and ‘Keep Largest Region’ isolated the tibia. A filled mask minus the marrow cavity yielded a cortical tissue mask. Intracortical canals were identified by subtracting the binary tibia image, excluding osteocyte lacunae due to resolution limits [42]. BoneJ's ‘Particle Analyzer’ quantified cortical porosity [43].

### 2.5 Three‐point Bending

Left femurs from 6‐week‐old animals (n = 5–7/genotype/sex) were cleaned of soft tissue and frozen in distilled water at −20°C. Three‐point bending was carried out on thawed femurs using a LRX5 materials testing machine (Lloyd Instruments) fitted with a 100N load. The span was fixed at 10 mm, and bones loaded with a crosshead speed of 1 mm/min until failure [36]. Load‐extension curves were generated to identify maximum load. Stiffness and yield were calculated from the slope of the linear region of the load‐extension curve using a polynomial fit.

### 2.6 Histological Analysis

Right hindlimbs were fixed for 24 h in 4% paraformaldehyde before being decalcified in 10% ethylenediaminetetraacetic acid for 21 days at 4°C, and wax‐embedded. Coronal sections (6 µm) were stained with Haematoxylin and Eosin, Safranin O/Fast green, and Toluidine blue using standard procedures. Immunohistochemical analysis was performed using anti‐MMP13, SOX9, OPN and ENPP1 (Table S2). Briefly, sections were dewaxed in xylene and rehydrated. For antigen retrieval, sections were incubated at 37°C for 30 min in 1 mg/mL trypsin (MMP13, OPN, ENPP1) or 10 mm sodium citrate at 60°C for 90 min (SOX9). Endogenous peroxidases were blocked by treatment with 0.3% H_2_O_2_ in methanol for 30 min at room temperature. The Vectastain ABC universal detection kit (Vector Laboratories) was used according to the manufacturer's instructions, with diaminobenzidine (DAB) solution used to detect the location of antigens. The sections were finally dehydrated and mounted in DPX. The height of the growth plate proliferating and hypertrophic zones was measured at 5 different points based on established cell morphology [44].

For methyl methacrylate (MMA) histology, samples were dehydrated through an alcohol series and two changes of xylene at 4°C using a Leica ASP 300 tissue processor. The samples were infiltrated at 4°C under vacuum with a mixture of 88.99% MMA, 10% dibutyl phthalate, 1% Perkadox 16, and 0.01% Tinogard TT for 5 days. After infiltration, the samples were placed in Teflon embedding blocks and left to polymerize in a water bath at room temperature for a minimum of 2 days. The lids were subsequently removed, and embedding rings were attached using Technovit 3040. Blocks were left to harden for at least 2 days before sectioning at 5 µm using a Leica RM2265 motorized microtome fitted with a tungsten steel D‐profile knife. The sections were mounted on X‐tra adhesive‐coated microscopy slides, covered with kissol film, and placed under pressure in a slide‐press at 37°C for > 48 h. Tartrate‐resistant acid phosphatase (TRAP) and von Kossa staining were conducted as described previously, and sections were analyzed using the bone histomorphometry software, HistoMorph [45].

### 2.7 Raman Spectroscopy

Raman spectra were acquired using an InVia Qontor Raman microscope (Renishaw, UK) equipped with a 633 nm HeNe laser and 1800 lines/mm grating (providing a spectral resolution of ∼1 cm^−1^) and a Master Renishaw Centrus 1UTR51 charge‐coupled device (CCD). Prior to data acquisition, CCD and spectrometer slits were aligned using the auto‐align function, and the laser spot was manually centered on the crosshairs using the camera. The system was intensity‐ and wavenumber‐calibrated to the 520.7 cm^−1^ peak of an internal silicon substrate. Single static point scans with an exposure time of 20 s, 3 accumulations and 100% laser power (∼31 mW at the sample) between 650–1750 cm^−^^1^ within the “Fingerprint region” [46] were set using the WiRE 5.6 software (Renishaw, UK) and were acquired using a 20x air objective (NA of 0.4) yielding a diffraction limited spot size of ∼965 nm. A total of 75 spectra, presented as class means, were obtained from randomly selected locations from the tibial epiphysis in MMA‐embedded sections (n = 3/genotype/sex).

Cosmic ray artefacts were removed from individual spectra upon acquisition in WiRE 5.6, as previously described [47]. Subsequent spectral preprocessing and analysis were performed in iRootLab [48]. Spectra were wavelet denoised (Haar wavelets, 6‐point smoothing) prior to the fitting of a fifth‐order polynomial for background fluorescence removal. Spectra were anchored to the x‐axis using the rubber‐band function and vector‐normalized. The second‐order derivative was calculated ahead of univariate spectral deconvolution in WiRE 5.6. The Raman peak corresponding to PMMA was identified at ∼812 cm^−1^ as assigned by [49]. The peak positions corresponding to the major vibrations of ECM components, including proline (853 cm^−1^), hydroxyproline (876 cm^−1^), hydroxyapatite (960 cm^−1^), and B‐type carbonate (1070 cm^−1^) were identified in second order derivative spectra. Peak areas were extracted by spectral deconvolution, wherein a mixture of Lorentzian and Gaussian curves was fitted to class mean spectra for specified Raman bands of interest.

### 2.8 Backscattered SEM

Right humeri were fixed in 4% paraformaldehyde, 2.5% glutaraldehyde (in 0.1 m sodium cacodylate) and stored in fixative at 4°C [50]. Samples were bisected, dehydrated in an ethanol series, and embedded in epoxy resin (TAAB), and the surface polished using sequential grade diamond polishing strips (Agar Scientific). The block was mounted on a 25 mm stub, sputter‐coated with 10 nm Platinum using a Q150T ES coater (Quorum Technologies), and examined under back‐scattered SEM (BSE‐SEM) using a TESCAN Clara 2 field emission gun SEM equipped with a low‐energy annular backscattered detector and operating at 10 keV, at a working distance of 10 mm.

### 2.9 Western Blotting

Femoral bone tissues were homogenized using a Rotor‐Stator Homogenizer (IKA Ultra‐Turrax T10) in radioimmunoprecipitation assay (RIPA) buffer containing protease inhibitors (Roche). Protein concentrations were measured by a BCA protein assay kit. The extracted proteins were equally loaded and separated using a 4–12% Bis‐Tris protein gel and then transferred to a Polyvinylidene fluoride (PVDF) membrane with a semi‐dry transfer technique. The non‐specific sites of protein on the membrane were blocked with Intercept (TBS) Blocking Buffer (LI‐COR Biosciences, Ltd.). Membranes were incubated sequentially with primary antibodies overnight at 4°C and with secondary antibodies for 1 h at room temperature (Table S2). β‐actin conjugated with Horseradish Peroxidase (HRP) was incubated for 20 min at room temperature. The specific protein signals were detected by the ultra‐sensitive enhanced chemiluminescence (ECL) substrates and imaged by the GeneGnome XRQ chemiluminescence imaging system (Syngene).

### 2.10 Serum Pyrophosphate (PP_i_) and Alkaline Phosphatase (ALP) Detection

Plasma was collected by centrifugation at 2500 × *g* for 10 min at 4°C following blood clotting for 30 min at room temperature. PP_i_ levels were measured using a colorimetric PP_i_ assay kit (Abcam) according to the manufacturer's instructions. ALP was detected using a colorimetric ELISA kit (Novus Biologicals), as per the manufacturer's instructions. Absorbance was measured using a spectrophotometer (Biochrom EZ Read 2000) at 570 nm (PP_i_) or 450 nm (ALP).

### 2.11 Quantitative Polymerase Chain Reaction (qPCR) and PCR Array

Left femora were cleaned of soft tissue, bone marrow removed by centrifugation, and frozen at −80°C. Bones were thawed and homogenized using a Rotor‐Stator Homogenizer (IKA Ultra‐Turrax T10) in ice‐cold Qiazol reagent. RNA was isolated and purified using a Qiagen RNeasy mini kit, according to the manufacturer's instructions.

RNA (2 µg) was reverse transcribed using a Qiagen QuantiTect RT kit, according to the manufacturer's instructions. The PCR was performed using cDNA (50 ng), a Brilliant II SYBR Green mastermix (Agilent), and run on an Agilent AriaMx PCR machine. Gene expression data for the qPCR were normalized to *β‐actin* and *Atbp5* using the geometric mean and analyzed using the ΔΔCt method [51]. Primer sequences can be found in Table S3.

### 2.12 Statistical Analysis

Analyses were performed using GraphPad Prism software (version 10.1.2). A one‐way ANOVA followed by Tukey's post hoc analysis was used to test for significance following normality testing using the Shapiro‐Wilk test. Results are presented as the mean ± standard error of the mean (SEM).

For the cortical bone analysis, R software (version 4.2.2) was used to generate line graphs and perform statistical analyses. Data were normally distributed, as assessed using the Shapiro‐Wilk normality test. For comparison between genotypes, a one‐way ANOVA followed by Tukey's post hoc analysis was used to test for significance. Lines represent the mean, with shading representing ± SEM. Color heatmaps display the statistical significance at matched locations along the graph, showing the genotype interaction and comparisons between genotypes. Red = *p* < 0.001, green = *p* < 0.01, yellow = *p* < 0.05, grey = *p*> 0.05 (not significant).

## 3 Results

### 3.1 Dual Deletion of *Alpl* and *Phospho1* Results in Complete Lack of Long Bone Mineralization in Postnatal Male and Female Mice

Mice with a *Prx1*‐specific deletion of TNAP were bred onto a global *Phospho1* ^−/−^ background (*Alpl^Prx1/Prx1^;Phospho1^−/−^*). litters also consisted of wildtype (WT), *Prx1*‐specific TNAP knockouts (*Alpl^Prx1/Prx1^*), *Phospho1* global knockouts (*Phospho1^−/−^*) and mice carrying a heterozygous *Prx1‐*specific TNAP deletion on a *Phospho1*‐null background (*Alpl^wt/Prx1^;Phospho1^−/−^*).

**FIGURE 1:**
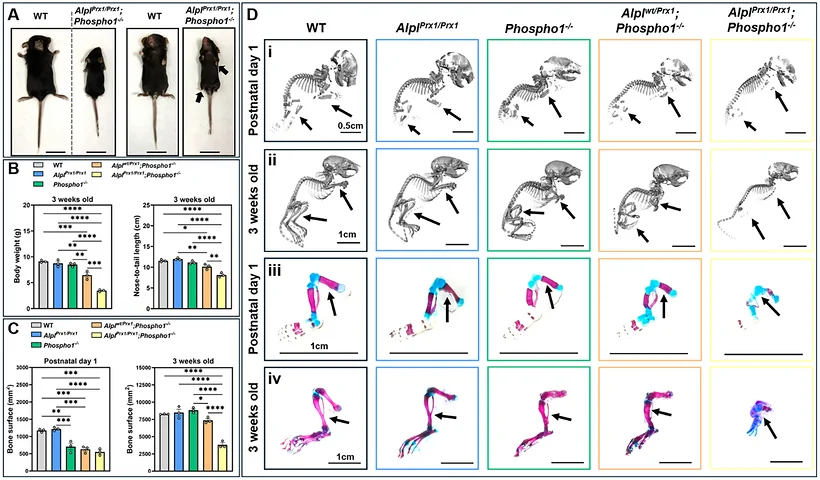
Gross phenotyping of mice with deletions in *Alpl* and *Phospho1* at postnatal day 1 and 3‐weeks of age highlight severe malformations of the limbs and reduced size. (A) Back and front views of WT and *Alpl^Prx1/Prx1^;Phospho1^−/−^* mice at 3‐weeks of age. Scale bar = 2 cm. (B) Body weight and nose‐to‐tail lengths in 3‐week‐old mice. (C) Bone surface area of the whole skeleton analysed by µCT at postnatal day 1 and at 3‐weeks old. (D) Representative µCT 3D‐renderings of whole skeletons at (i) postnatal day 1 (scale bar = 5 mm) and (ii) 3 weeks old (scale bar = 1 cm). Arrows indicate forelimbs and hindlimbs in all genotypes, with *Alpl^Prx1/Prx1^;Phospho1^−/−^* mice lacking mineralized limbs. Whole mount imaging of hindlimbs in (iii) postnatal day 1 and (iv) 3 weeks old. Arrows indicate mineralization in all genotypes except for *Alpl^Prx1/Prx1^;Phospho1^−/−^* mice. Scale bar = 1 cm. Data are presented as mean ± SEM with points showing individual animals (n = 3/genotype/age). ^*^ = *p* < 0.05, ^**^ = *p* < 0.01, ^***^ = *p* < 0.001, ^****^ = *p* < 0.0001.

Western blotting indicated that the gene targeting strategy was successful with the expected absence of TNAP and/or PHOSPHO1 protein in the limbs of the various knockout mice (Figure S1). *Alpl^Prx1/Prx1^;Phospho1^−/−^* mice of both sexes were significantly smaller than their littermates, with severe limb and paw malformations evident at 3‐weeks of age (Figure 1A). No gross visual differences were observed between the other genotypes (*Alpl^Prx1/Prx1^, Phospho1^−/−^, Alpl^wt/Prx1^;Phospho1^−/−^)* and WT mice. Whilst *Alpl^wt/Prx1^;Phospho1^−/−^* mice similarly weighed less and were smaller than WT, *Alpl^Prx1/Prx1^* and *Phospho1^−/−^* animals (*p* < 0.05); *Alpl^Prx1/Prx1^;Phospho1^−/−^* mice were significantly smaller than all other genotypes at 3‐weeks of age (*p* < 0.001; Figure 1B).

Skeletal analysis by micro‐computed tomography (µCT) at postnatal day 1 (PN1) showed that in comparison to WT and *Alpl^Prx1/Prx1^* mice, deletion of *Phospho1* in all other genotypes resulted in reduced bone surface area (*p* < 0.01; Figure 1C). The lack of mineralized limbs in *Alpl^Prx1/Prx1^;Phospho1^−/−^* mice is evident in 3D‐rendered images, whereas long bones and digits are observed in all other genotypes, but to a lesser extent in *Phospho1^−/−^* and *Alpl^wt/Prx1^;Phospho1^−/−^* mice (Figure 1D‐i). By 3‐weeks of age, the mineralized surface area of *Phospho1^−/−^* mice had recovered, whereas *Alpl^wt/Prx1^;Phospho1^−/−^* mice maintained this reduction (*p* < 0.05) and was further reduced in *Alpl^Prx1/Prx1^;Phospho1^−/−^* mice (*p* < 0.0001; Figure 1C, D‐ii). Histological staining of skeletal elements from PN1 and 3‐week‐old mice with alcian blue and alizarin red confirmed a virtual absence of mineralized bone in *Alpl^Prx1/Prx1^;Phospho1^−/−^* hindlimbs (Figure 1D‐iii, iv and Figure S2). Visible limb malformations, including bowing and dysplasia, were observed in *Phospho1^−/−^* animals, and to a greater extent in *Alpl^wt/Prx1^;Phospho1^−/−^* mice (Figure 1D‐ii–iv and Figure S2).

**FIGURE 2:**
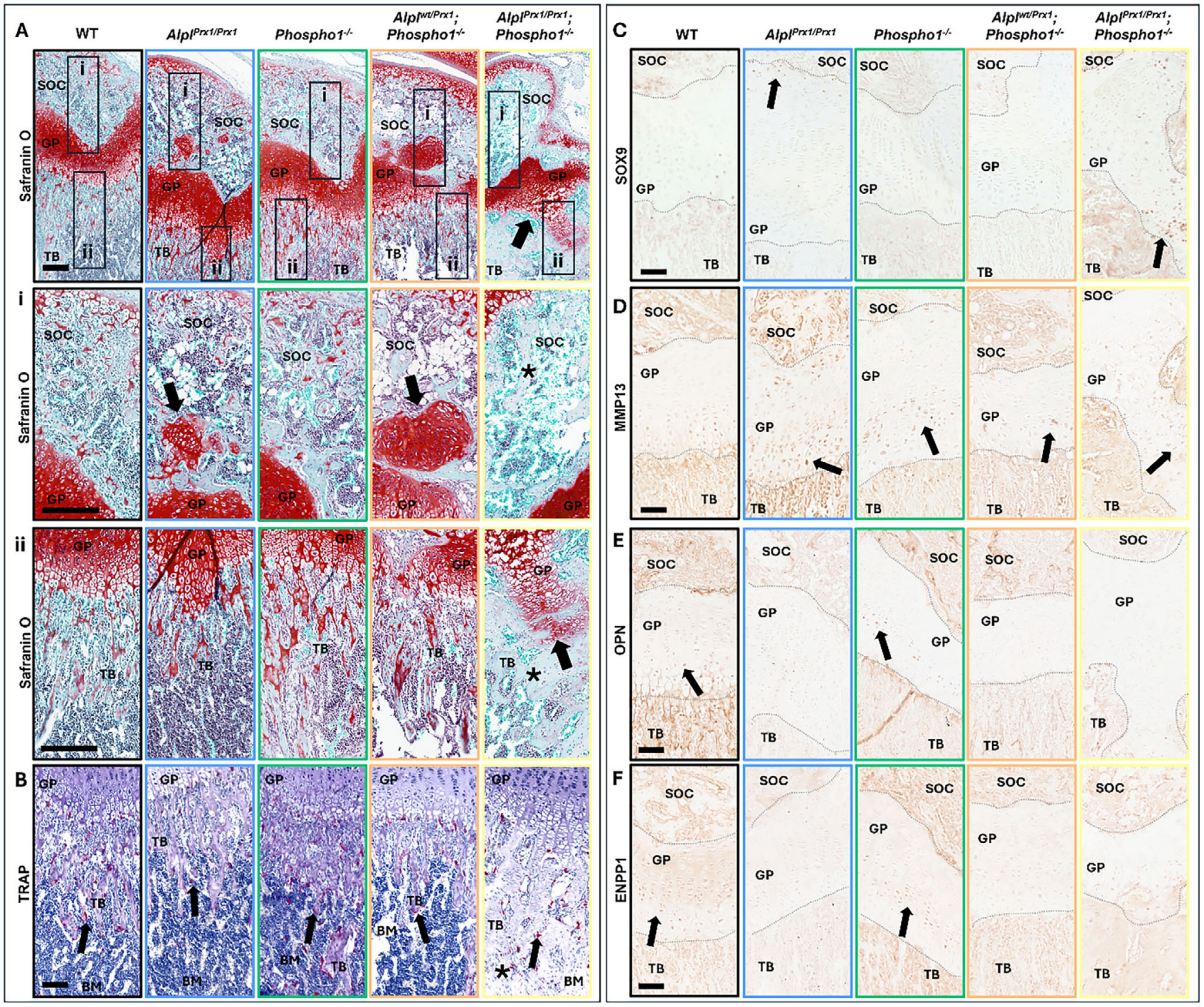
Histological analysis of 3‐week‐old mice with deletions in *Alpl* and *Phospho1* reveals abnormal bone formation in the secondary ossification centres, enlarged peripheral growth plate regions and a reduction in osteoclasts. (A‐i) Representative images of Safranin‐O/Fast green staining of femurs from wild‐type (WT), *Alpl^Prx1/Prx1^*, *Phospho1^−/−^, Alpl^wt/Prx1^;Phospho1^−/−^* and *Alpl^Prx1/Prx1^;Phospho1^−/−^* mice. Images with a higher magnification of the SOC and TB are shown in A‐i, ii. Arrows indicate accumulations of hypertrophic chondrocytes in the SOC and extension into the metaphysis. ^*^ indicate non‐mineralized structures. Scale bar = 200 µm. (B) Representative TRAP labelling of osteoclasts. Arrows indicate osteoclast expression in all animals, albeit reduced with dual *Alpl* and *Phospho1* deletion. Representative (C) SOX9, (D) MMP13 (E) ENPP1 and (F) OPN immunolabelling. Arrows indicate chondrocytes expression in the GP and metaphyseal region. The different regions are delineated by a dashed line. Scale bar = 100 µm. Images are representative of n = 3/genotype/age. Scale bars for all images = 100 µm. GP = growth plate. SOC = secondary ossification centres, TB = metaphyseal trabecular bone.

Further histological analyses of hindlimbs from 3‐week‐old old mice using Safranin O for proteoglycans and glycosaminoglycans revealed a lack of bone formation in the secondary ossification centre (SOC) of *Alpl^Prx1/Prx1^*, *Alpl^wt/Prx1^;Phospho1^−/−^* and *Alpl^Prx1/Prx1^;Phospho1^−/−^* mice, driven by *Alpl* loss (Figure 2A). Here, accumulations of hypertrophic chondrocytes surrounded by matrix persist (Figure 2A, arrows) while the structure and composition of the articular cartilage appeared normal in all mice. Metaphyseal tissue beneath the growth plate was devoid of clear trabeculae in *Alpl^Prx1/Prx1^;Phospho1^−/−^* mice with instead, non‐mineralized structures and minimal bone marrow evident (Figure 2A,B). Dual deletion of *Alpl* and *Phospho1* also resulted in regionalized extension of the growth plate cartilage into this metaphyseal region (Figure 2A and Figure S2C,D), containing SOX9‐ and MMP13‐expressing chondrocytes (Figure 2C,D). Further examination of the growth plate cartilage revealed a reduction in OPN and ENPP1 with loss of *Alpl* (Figure 2E,F). TRAP‐positive osteoclasts were present at the osteochondral junction of all mice (Figure 2B), however quantification revealed a significant decrease in osteoclast surface/bone surface (Oc.S/B.S) and number of osteoclasts/bone volume (N. OC/BV) in *Alpl^Prx1/Prx1^;Phospho1^−/−^* in comparison to WT mice (*p* < 0.01; Table 1).

**TABLE 1:** Quantification of TRAP‐labeled osteoclasts in the metaphysis of 3‐week‐old mice. Data represented as mean ± SEM (*n* = 3/genotype) for osteoclast surface/bone surface (Oc.S/B.S) and number of osteoclasts/bone volume (N. Oc/BV). a = vs. WT (*p* < 0.01), b = vs. *Alpl^Prx1/Prx1^* (*p* < 0.05), c = vs. *Phospho1^−/‒^* (*p* < 0.01).

	WT	*Alpl^Prx1/Prx1^*	*Phospho1^−/−^*	*Alpl^wt/Prx1^;* *Phospho1^−/−^*	*Alpl^Prx1/Prx1^;* *Phospho1^−/−^*
Oc.S/B.S (%)	14 ± 1.8	11 ± 1.4	8.5 ± 1.1	6.9 ± 1.9	4.1 ± 0.95^a)^
N.Oc/BV (mm^−2^)	547 ± 35	466 ± 49	499 ± 32	407 ± 79	188 ± 35 ^abc^

### 3.2 At Skeletal Maturity, One Functional *Alpl* Allele Rescues Biomineralization and the Epiphyseal Defects Observed With Dual Phosphatase Deletion

Although viable, *Alpl^Prx1/Prx1^;Phospho1^−/−^* mice did not thrive as well as their littermates post‐weaning and remained stunted in their growth. In accordance with humane animal research practices in the UK, these mice were therefore not maintained beyond 3‐weeks of age. Subsequent analyses were conducted on 6‐week‐old *Alpl^wt/Prx1^;Phospho1^−/−^* heterozygous mice to allow delineation of the individual and dual roles of these phosphatases in the maturing skeleton.

Male and female mice of each genotype had comparable gross anatomy (Figure S3A) and serum ALP and PP_i_ levels (Figure S3B). However, while *Alpl^Prx1/Prx1^* mice had similar body weight and lengths as WTs, *Phospho1^−/−^* mice were smaller than WT mice (*p* < 0.05; Table S1). *Alpl^wt/Prx1^;Phospho1^−/−^* mice were also smaller in length than WTs, but body weight was comparable (Table S1). Analysis of bone lengths by µCT revealed that all genotypes (except *Alpl^Prx1/Prx1^* males) had shorter tibias than WT mice, with progressively shorter bones in *Alpl^Prx1/Prx1^* > *Phospho1^−/−^* > *Alpl^wt/Prx1^;Phospho1^−/−^* mice (*p* < 0.05; Table S1).

**FIGURE 3:**
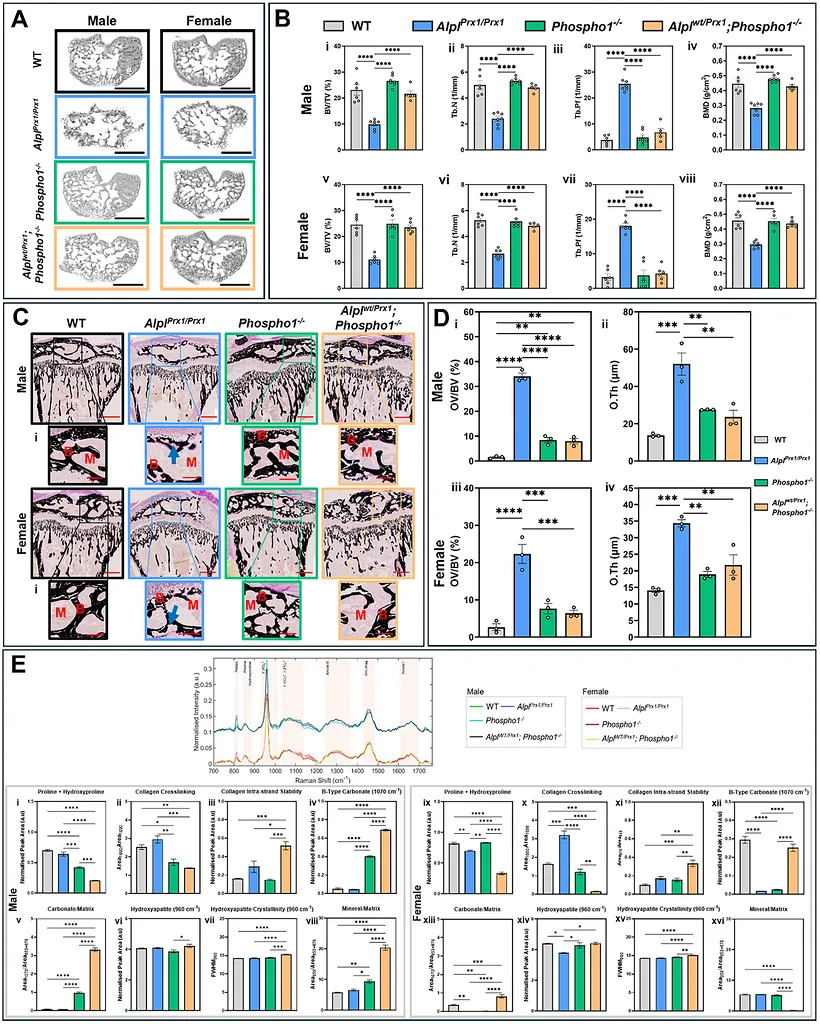
Tibial epiphyseal bone defects observed in 6‐week‐old mice with *Alpl* deletion and altered ECM composition in all genotypes. (A) Representative 3D‐reconstructed µCT images of the epiphyseal trabecular region in wild‐type (WT), *Alpl^Prx1/Prx1^*, *Phospho1^−/−^* and *Alpl^wt/Prx1^;Phospho1^−/−^* male and female mice. Scale bar = 1 mm. (B) Epiphyseal analysis of (i & v) bone volume fraction (BV/TV), (ii & vi) trabecular number (Tb.N), (iii & vii) trabecular pattern factor (Tb.Pf) and (iv & viii) bone mineral density (BMD) in males and females (n = 5‐7/genotype/sex). (C) Representative von Kossa‐stained images of the epiphyseal region in male and female mice. Arrows indicate areas of increased osteoid. B = bone, M = bone marrow. Scale bar = 500 µm; (i = 200 µm. (D) Quantification of (i & iii) osteoid volume/bone surface (OV/BV) and (ii & iv) osteoid thickness (O.Th) in the epiphyseal region of males and females, respectively (n = 3/genotype/sex). Data are presented as mean ± SEM with points showing individual animals. (E) Deviations in vector normalized class means (n = 75 spectra from 3 mice/genotype/sex) were detected in Raman bands corresponding to the ECM (proline, 853 cm^−1^; hydroxyproline, 876 cm^−1^; amide III region, 1242 cm^−1^; CH_2_ wag, 1450 cm^−1^ and amide I, 1660 cm^−1^) and mineral components (hydroxyapatite, 960 cm^−1^ and B‐type carbonate, 1070 cm^−1^) of the tibial epiphyses. Spectral deconvolution of Raman bands in components of (i & ix) type I collagen (proline + hydroxyproline), (ii & x) collagen crosslinking, (iii & xi) collagen intra‐strand stability, (iv & xii) B‐type carbonate, (v & xiii) carbonate/matrix ratio, (vi & xiv) hydroxyapatite mineralization and (vii & xv) crystallinity, and (viii & xvi) mineral/matrix ratio in males and females, respectively. Data are presented as the mean normalized peak area or full‐width half maximum ± SEM. * = *p* < 0.05, ** = *p* < 0.01, *** = *p* < 0.001, **** = *p* < 0.0001.

The observed defects in the SOC of 3‐week‐old mice prompted further investigations of this region in 6‐week‐olds. Here, defects were only observed in *Alpl^Prx1/Prx1^* mice. Morphometric analysis of the epiphyseal trabecular bone revealed reduced bone volume fraction (BV/TV), bone mineral density (BMD) and bone surface parameters in *Alpl^Prx1/Prx1^* mice (*p* < 0.0001, Figure 3A,B and Figure S4). Additional microarchitectural defects in the epiphysis including fewer trabeculae (Tb.N), increased spacing (Tb.Sp) and reduced complexity (Tb.Pf, Conn. Dn, Fractal dimension) were observed (*p* < 0.001, Figure 3A,B and Figure S4). Von Kossa staining demonstrated that this reduction in mineralization was accompanied by an increase in osteoid volume to bone volume (OV/BV) and thickness (O.Th) (*p* < 0.01, Figure 3C,D). Raman spectroscopy revealed alterations in epiphyseal ECM composition occur exclusively in female *Alpl^Prx1/Prx1^* mice, as indicated by reductions in type I collagen content (sum peak area of proline and hydroxyproline) and enhanced collagen crosslinking, which was associated with low levels of B‐type carbonate and modest reductions in hydroxyapatite (*p* < 0.05, Figure 3E and Figures S5 and S6).

Further sex differences were evident in the ECM of *Phospho1*‐deficient mice. Increased carbonation in both *Phospho1^−/−^* and *Alpl^wt/Prx1^;Phospho1^−/‒^* males was accompanied by reduced type I collagen content and crosslinking (Figure 3E and Figures S5 and S6). In female *Phospho1^−/−^* mice, however, a sex‐specific reduction in matrix carbonation occurred independently of changes to collagen and hydroxyapatite composition, whereas no change in carbonation was observed in female *Alpl^wt/Prx1^;Phospho1^−/‒^* animals, yet collagen crosslinking was impaired. Despite these differences, and in contrast to *Alpl^Prx1/Prx1^* mice, *Phospho1* deletion had no effect on any epiphyseal morphometric parameters or von Kossa staining of mineralised tissue in either sex (Figure 3A–D and Figure S4). Intriguingly, *Alpl^wt/Prx1^;Phospho1^−/−^* mice showed limited mineralization abnormalities in the epiphysis in comparison to WT animals (Figure 3A–D and Figure S4), with the only significant difference observed in OV/BV in male animals (Figure 3D, *p* < 0.01), suggesting a potential correction of the observed histological differences at 3‐weeks of age.

**FIGURE 4:**
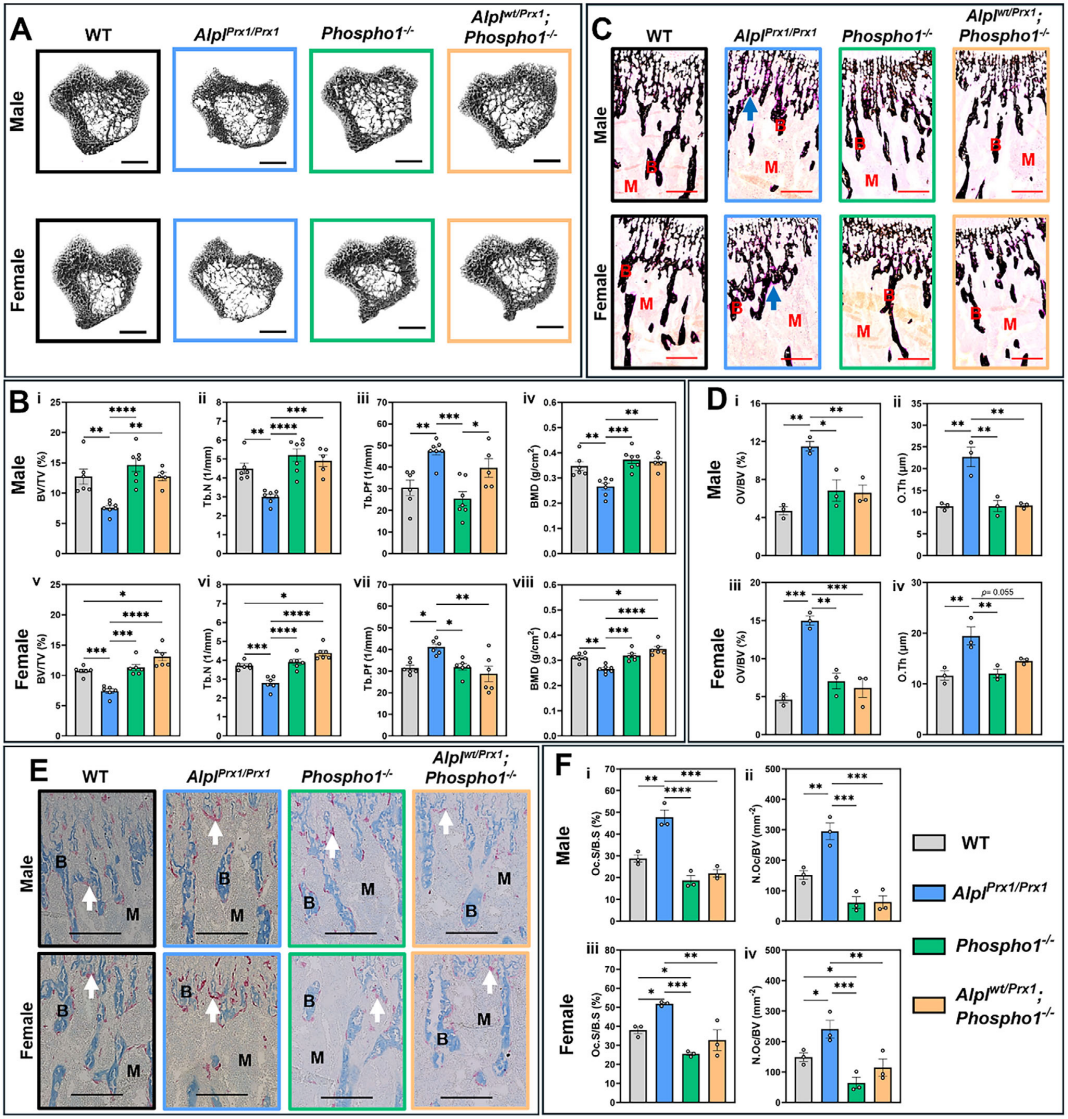
Tibial metaphyseal region analysis reveals trabecular compartment defects with *Alpl* deletion in 6‐week‐old mice, coupled with mineralization defects and increased osteoclasts. (A) Representative 3D‐reconstructed µCT images of the metaphyseal trabecular region in wild‐type (WT), *Alpl^Prx1/Prx1^*, *Phospho1^−/−^* and *Alpl^wt/Prx1^;Phospho1^−/−^* male and female mice. Scale bar = 1 mm. (B) Trabecular analysis of (i & v) bone volume fraction (BV/TV), (ii & vi) trabecular number (Tb.N), (iii & vii) trabecular pattern factor (Tb.Pf) and (iv & viii) bone mineral density (BMD) in males and females, respectively (n = 5‐7/genotype/sex). (C) Representative von Kossa‐stained images of the trabecular region. Arrows indicate areas of increased osteoid. B = bone, M = bone marrow. Scale bar = 200 µm. (D) Quantification of metaphyseal trabecular (i & iii) osteoid volume/bone volume (OV/BV) and (ii & iv) osteoid thickness (O.Th) in males and females, respectively (n = 3/genotype/sex). (E) Representative images of TRAP labelling in the metaphyseal region. Arrows indicate red‐labelled osteoclasts. B = bone, M = bone marrow. Scale bar = 200 µm. (F) Quantification of metaphyseal (i & iii) osteoclast surface/bone surface (Oc.S/B.S) and (ii & iv) number of osteoclasts/bone volume (N. Oc/BV) in male and female mice, respectively (n = 3/genotype/sex). Data are presented as mean ± SEM with points showing individual animals. ^*^ = *p* < 0.05, ^**^ = *p* < 0.01, ^***^ = *p* < 0.001, ^****^ = *p* < 0.0001.

The metaphyseal trabecular regions of *Alpl^Prx1/Prx1^* mice were also deleteriously affected, with reductions in BV/TV and BMD noted alongside disrupted trabecular microarchitecture (*p* < 0.05; Figure 4A,B and Figure S7). Osteoid mineralization was also impaired in these animals (*p* < 0.01; Figure 4C,D), which may be driven by increased osteoclast number and associated bone (re)modelling (*p* < 0.05; Figure 4E,F). Whilst morphometric analyses in this region of *Phospho1^−/−^* mice were unchanged (Figure 4A‐B), an increase in trabecular parameters was observed exclusively in female *Alpl^wt/Prx1^;Phospho1^−/‒^* mice (*p* < 0.05; Figure 4B‐v–viii and Figure S7). Despite conservation of osteoid levels, a trend toward reduced osteoclast number and surface was evident in male and female *Phospho1^−/‒^* and *Alpl^wt/Prx1^;Phospho1^−/−^* mice, although this did not reach statistical significance (Figure 4C–F).

**FIGURE 5:**
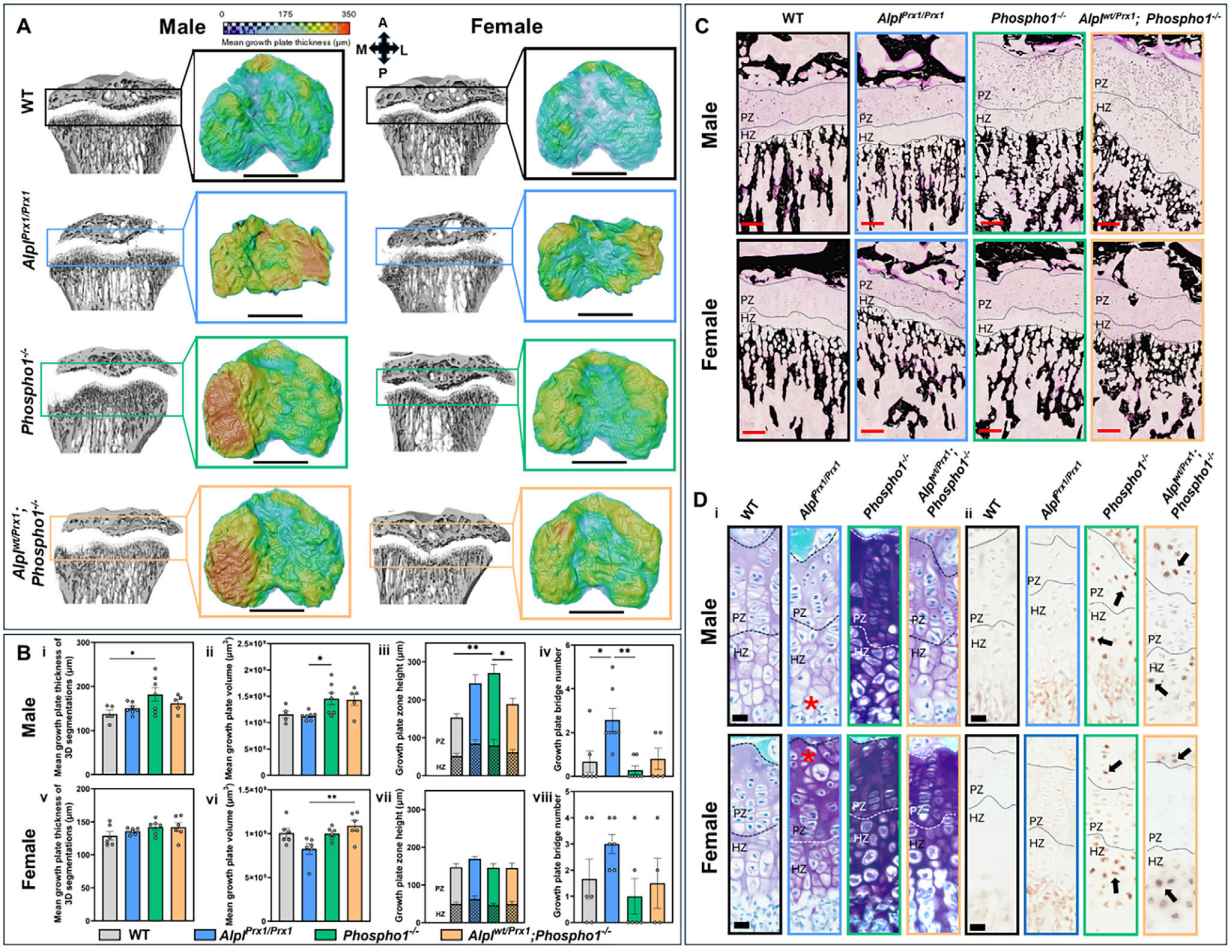
At 6 weeks of age, all genotypes display altered growth plate morphology. (A) Representative 3D‐rendered images of the segmented region of the growth plate from wild‐type (WT), *Alpl^Prx1/Prx1^*, *Phospho1^−/−^* and *Alpl^wt/Prx1^;Phospho1^−/−^* male and female mice. Scale bar = 1 mm. (B) Measurements of the (i & v) mean growth plate thickness and (ii & vi) volume of the 3D segmentations in A, (iii & vii) growth plate zone height, and (iv & viii) number of bridges in males and females, respectively (n = 5‐7/genotype/sex). (C) Representative von Kossa‐stained images of the growth plate. Proliferative (PZ) and hypertrophic (HZ) growth plate zones are delineated. Scale bar = 100 µm. (Di) Representative Toluidine Blue‐stained images of the growth plate and (ii) SOX9 immunolabelling in males and females. Proliferative (PZ) and hypertrophic (HZ) growth plate zones are delineated. * indicates enlarged chondrocytes. Arrows indicate SOX9 positive chondrocytes. Scale bar = 100 µm. Images are representative of n = 3/genotype/age. Data are presented as mean ± SEM with points showing individual animals. * = *p* < 0.05, ** = *p* < 0.01.

3D reconstructions of *Alpl^Prx1/Prx1^* growth plates further revealed malformation of the SOC (Figure 5A,B). Here, thickness and volume of these reconstructions, alongside growth plate zone height, were unchanged, while elevations in growth plate bridge number in males was observed (Figure 5B,C). This was accompanied by the accumulation of enlarged and disorganized chondrocytes in both the proliferative and hypertrophic regions (Figure 5D‐i). In contrast, male *Phospho1^−/−^* mice produced thicker growth plates, predominantly on the medial aspect of the condyle (Figure 5A–C). This was linked to an expansion of the growth plate, where the formation of ‘pit‐like’ regions containing disorganized, SOX9‐expressing hypertrophic chondrocyte populations were evident (Figure 5A,D‐ii). These trends were also observed in male *Alpl^wt/Prx1^;Phospho1^−/−^* mice, yet the pit‐like structures were not observed in females of either genotype.

### 3.3 Deletion of *Phospho1* Results in Tibial Geometry Changes, Which are Compounded Upon the Additional Deletion of One *Alpl* Allele

**FIGURE 6:**
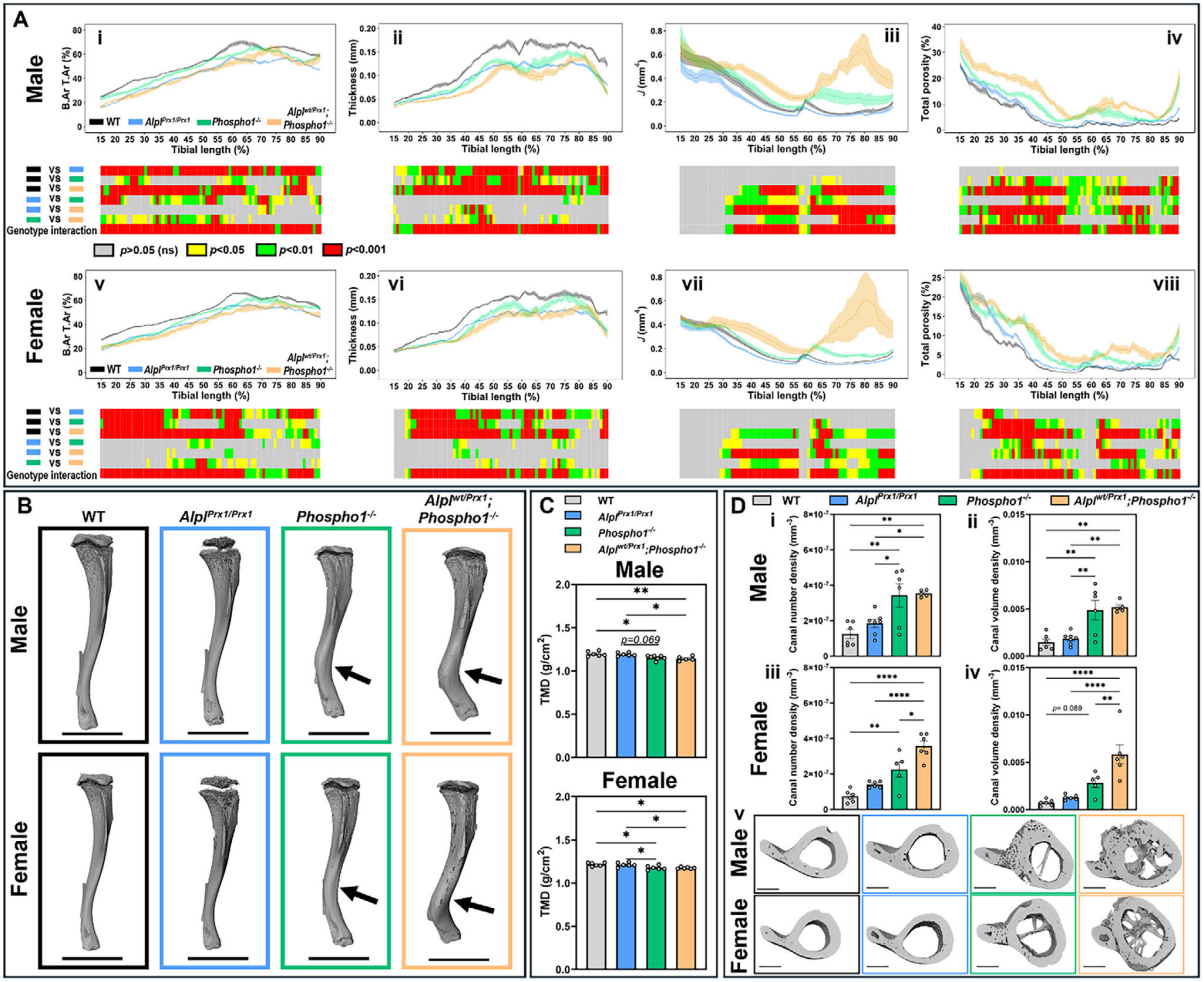
In 6‐week‐old mice, all genotypes exhibit reduced tibial cortical bone area and thickness, but *Phospho1* deficient animals have severely altered geometry and a mineralization defect. (A) Measurement and statistical analysis heat map for (i & v) bone area / tissue area (B. Ar T. Ar), (ii & vi) thickness, (iii & vii) predicted resistance to torsion (*J*), and (iv & viii) total porosity in male and female mice, respectively. Line graphs represent mean ± SEM for wild‐type (WT; black), *Alpl^Prx1/Prx1^* (blue), *Phospho1^−/−^* (green) and *Alpl^wt/Prx1^;Phospho1^−/−^* (orange) male and female mice (n = 5‐7/genotype/sex). Graphical heat map summarizes statistical differences at specific matched locations along the tibial length (15–90%). Red  =  *p* < 0.001, green  =  *p* < 0.01, yellow  =  *p* < 0.05, grey  =  *p* > 0.05 (not significant). (B) Representative 3D‐rendered µCT images of the entire tibia. Arrows indicate bowing of the limb. Scale bar = 0.5 cm. (C) Quantification of tissue mineral density (TMD, g/cm^2^) in male and female mice. (D) Quantification of (i&iii) canal density and (ii&iv) volume density at the tibiofibular junction in males and females, respectively. (v) Representative 3D‐reconstructed µCT images in males and females showing the porosity at the tibiofibular junction. Scale bar = 500 µm. Data are presented as mean ± SEM with points showing individual animals (n = 5‐7/genotype/sex). * = *p* < 0.05, ** = *p* < 0.01.

To assess the effect of *Alpl* and *Phospho1* deletion along the full tibial length, 2D whole bone analysis and three‐point bending were performed. Compared to WT mice, cortical bone area fraction (B.Ar/T.Ar) and thickness of male and female mice were reduced in all genotypes (Figure 6A), leading to detrimental effects on the tibial mechanical properties in *Alpl^Prx1/Prx1^* mice of both sexes, female *Phospho1^−/−^*, and female *Alpl^wt/Prx1^;Phospho1^−/−^* animals (Table 2). Parameters associated with bone shape (predicted resistance to torsion (*J*), maximum and minimum moments of inertia (*I_max_* & *I_min_*)), and tissue and bone perimeter (T.Pm and B.Pm, respectively) were increased in *Phospho1^−/‒^* mice compared to WT and *Alpl^Prx1/Prx1^* (Figure 6A and Figure S8). In *Alpl^wt/Prx1^;Phospho1^−/−^* animals, these effects were compounded, particularly at the most distal end and likely due to the severe bowing observed in this region (Figure 6A,B). Whilst tissue mineral density (TMD) was also reduced in *Phospho1^−/‒^* and *Alpl^wt/Prx1^;Phospho1^−/−^* mice (*p* < 0.05; Figure 6C), male mice of these genotypes were unaffected by mechanical testing (Table 2). Total porosity was increased along the tibial length in *Phospho1^−/‒^* and *Alpl^wt/Prx1^;Phospho1^−/−^* male and female mice (Figure 6A‐iv–viii), whilst further analysis specifically at the tibiofibular junction identified increased canal and volume density (Figure 6D).

**TABLE 2:** Three‐point bending analysis of 6‐week‐old femurs from males and females. Data represented as mean ± SEM for *n* = 5‐7/genotype/sex. a = vs. *Alpl^Prx1/Prx1^* (*p* < 0.05), b = vs. *Alpl^Prx1/Prx1^* (*p* < 0.01), c = vs. *Alpl^wt/Prx1^;Phospho1^−/−^* (*p* < 0.05), d = vs. *Phospho1^−/−^* (*p* < 0.05), and e = vs. *Alpl^wt/Prx1^;Phospho1^−/−^* (*p* < 0.0001).

Male				
	WT	*Alpl^Prx1/Prx1^*	*Phospho1^−/−^*	*Alpl^wt/Prx1^;* *Phospho1^−/−^*
**Max Load (*N*)**	9.02 ± 0.82	6.66 ± 0.37	9.86 ± 0.94	10.53 ^a^ ± 1.57
**Stiffness (*N*/m)**	25.56 ^a^ ± 1.39	16.39 ± 1.54	22.72 ± 2.51	21.98 ± 3.74
**Yield (*N*)**	5.89 ^a^ ±0.96	2.74 ±0.44	6.09 ^a^ ± 0.55	6.91 ^b^ ± 1.32

Female				
	WT	*Alpl^Prx1/Prx1^*	*Phospho1^−^^/^^−^*	*Alpl^wt/Prx1^;* *Phospho1^−^^/^^−^*
**Max Load (*N*)**	7.44 ± 0.63	7.03 ± 0.39	8.20 ± 0.70	7.44 ± 0.76
**Stiffness (*N*/m)**	23.74 ^b, d, e^ ± 1.05	17.00 ± 0.57	18.00 ± 0.64	10.90 ^a, d^ ± 3.16
**Yield (*N*)**	4.70 ^c^ ± 0.49	4.06 ± 0.32	4.68 ^c^ ± 0.38	3.04 ± 0.33

**FIGURE 7:**
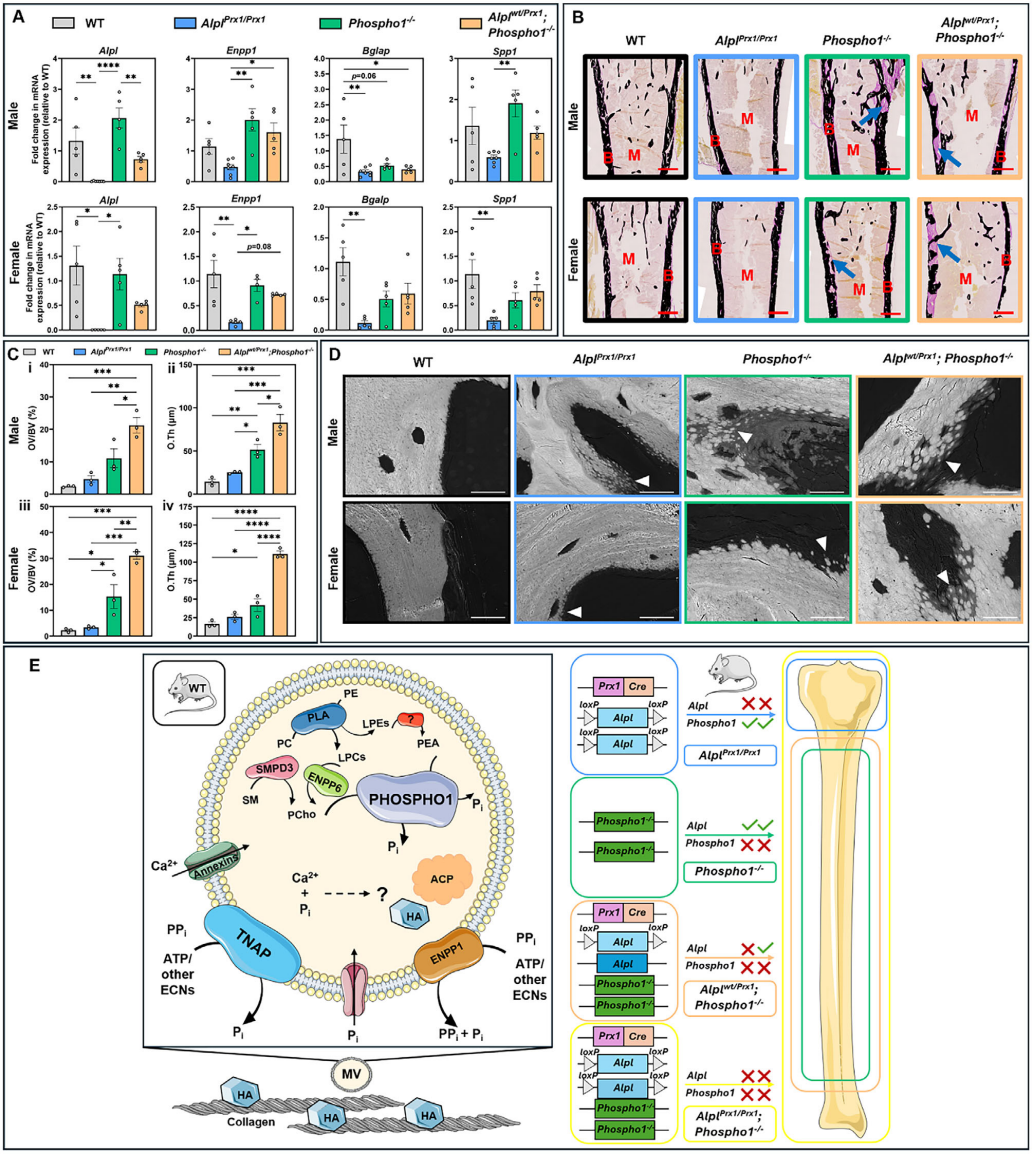
At 6‐weeks of age, *Alpl*‐deficient mice exhibit reduced mineralization gene expression, yet cortical mineralization defects are observed with *Phospho1* deletion and is compounded in *Alpl^wt/Prx1^;Phospho1^−/‒^* animals. (A) RT‐qPCR of key mineralization genes from femurs of wild‐type (WT), *Alpl^Prx1/Prx1^*, *Phospho1^−/−^* and *Alpl^wt/Prx1^;Phospho1^−/−^* male and female mice (n = 5‐7/genotype/sex). (B) Representative von Kossa‐stained images of the cortical region of the tibial diaphysis. Arrows indicate increased areas of osteoid in *Phospho1^−/−^* and *Alpl^wt/Prx1^;Phospho1^−/−^* mice. B = bone, M = bone marrow. Scale bar = 500 µm. (C) Quantification of (i & iii) osteoid volume/bone volume (OV/BV) and (ii & iv) osteoid thickness (O.Th) in the cortical region (n = 3/genotype/sex). (D) Back scattered scanning electron microscopy images of the cortices. Arrowheads indicate failure of mineralization foci to propagate within areas of hypomineralization. Scale bar = 20 µm. Images are representative of n = 3/genotype/age. (E) Schematic showing the synergistic roles of TNAP and PHOSPHO1 in the unified model of biomineralization and summary of our findings. Images provided by Servier Medical Art (https://smart.servier.com), licensed under CC BY 4.0 (https://creativecommons.org/licenses/by/4.0/). Abbreviations: ACP: Amorphous calcium phosphate; Ca^2+^: Calcium; ECN: extracellular nucleotides; HA: hydroxyapatite; LPC: lyso‐phosphatidylcholine; LPE: lyso‐phosphatidylethanolamine; MV: matrix vesicle; PC: phosphatidylcholine; PCho: phosphocholine; PE: phosphatidylethanolamine; PEA: phosphoethanolamine; P_i_: inorganic phosphate; PLA: phospholipase A2; PP_i_: inorganic pyrophosphate; SM: sphingomyelin. Data are presented as mean ± SEM with points showing individual animals. * = *p* < 0.05, ** = *p* < 0.01, *** = *p* < 0.001, **** = *p* < 0.0001.

To assess the mechanisms underpinning these alterations in cortical architecture and geometry, the expression of key genes involved in osteoblast differentiation and mineralization were investigated in femoral diaphyseal bone. Interestingly, expression of *Enpp1*, *Bglap* (Osteocalcin) and *Spp1* (OPN) were downregulated in *Alpl^Prx1/Prx1^* femurs compared to WT (*p* < 0.05; Figure 7A). Due to the nonsense mutation at amino acid 74 in exon 3 of *Phospho1^−/−^* mice, gene primers for *Phospho1* expression could only be used on *Alpl^Prx1/Prx1^* animals, which showed a reduction in *Phospho1* expression in both sexes (*p* < 0.05; Figure S9A). Genes associated with membrane PCho synthesis (*Chkb*, *Smpd3*) and phosphate transport (*Slc20a1, Slc20a2*) were generally elevated in *Phospho1^−/‒^* mice, whilst *Ank* and *Phex* were only increased in *Phospho1^−/−^* and *Alpl^wt/Prx1^;Phospho1^−/−^* males, respectively (*p* < 0.05; Figure S9A). No significant differences were observed in gene expression of markers of the Wnt signaling and RANK/RANKL/OPG pathway (Figure S9A). Despite gene expression changes being predominantly affected by *Alpl*‐loss, von Kossa staining of the cortical region revealed impaired mineralization and an increase in osteoid in *Phospho1^−/‒^* and *Alpl^wt/Prx1^;Phospho1^−/−^* male and female mice (Figure 7B,C). The presence of osteoid was most obvious in *Alpl^wt/Prx1^;Phospho1^−/−^* animals showing large areas of non‐mineralized cortical tissue. Back scattered scanning electron microscopy (BSSEM) of the cortices confirmed this aberrant mineralization in *Phospho1^−/‒^* and *Alpl^wt/Prx1^;Phospho1^−/‒^* mice, with these animals displaying hypomineralized regions, large voids and inconsistent patchy regions of osteoid, highlighting a clear mineralization defect (Figure 7D and Figure S9B).

## 4 Discussion

This is the first study to overcome the perinatal lethality of global dual TNAP and PHOSPHO1 deletion in vivo to define how these two phosphatases have a synergistic effect when they cooperate to form a fully mineralized postnatal skeleton [11]. Although PHOSPHO1 and TNAP have been individually implicated in biomineralization, whether they act redundantly, sequentially, or in spatially distinct pathways during postnatal bone development remains unresolved. Herein, we utilized *Prx1*‐*Cre* mice to target *Alpl* deletion in the early limb bud mesenchyme, including osteoblasts and chondrocytes, to generate *Alpl^Prx1/Prx1^;Phospho1^−/−^* mice [21, 52, 53]. Through constricting *Alpl* loss to the appendicular skeleton, our conditional knockout model exhibited mineralization of the central axis and therefore, unlike the single, stillborn, global *Alpl* and *Phospho1* double knockout mouse [11], survived postnatally. *Alpl^Prx1/Prx1^;Phospho1^−/−^* mice, however, were noticeably smaller than their littermates and, using a variety of multi‐modal approaches to conduct a full skeletal phenotyping of these mice, we identified a lack of mineralization in their limbs, resembling a non‐mineralized cartilaginous matrix. Ablation of each enzyme alone produced alterations to spatially discrete anatomical regions of the limb, with *Alpl^Prx1/Prx1^* mice displaying SOC and metaphyseal defects, whilst *Phospho1* deletion preferentially resulted in abnormalities to the cortical bone of the diaphysis. Collectively, these data provide evidence to support a mechanistic framework for complementary and sequentially dependent biomineralization, whereby mineral formation within the MV is driven by P_i_ production by PHOPSHO1 and the influx of P_i_ generated outside the vesicles by TNAP. Additionally, TNAP controls the extravesicular propagation of mineralization via PP_i_, which helps explain the spatial and developmental differences observed (Figure 7E) [1, 3, 37, 54]. The observation that a single *Alpl* allele in *Alpl^wt/Prx1^;Phospho1^−/−^* mice was able to partially rescue the defective skeletal mineralization of *Alpl^Prx1/Prx1^;Phospho1^−/−^* mice supports the gene‐dose‐linear model of TNAP activity where the skeletal phenotype changes in proportion to TNAP activity. Our findings provide vital insights into our understanding of hypo‐ and hyper‐mineralized pathologies, ultimately enabling future treatment strategies.

Previous studies have revealed spatial differences in TNAP and PHOSPHO1 expression, which may offer some explanation as to the preferential epiphyseal/metaphyseal defects observed in *Alpl^Prx1/Prx1^* mice, and the pronounced diaphyseal defects in *Phospho1* ^−/−^ mice observed herein [27, 63]. The exacerbated cortical phenotype in *Alpl^wt/Prx1^;Phospho1^−/−^* heterozygous mice further strengthens this. Indeed, TNAP expression was broadly expressed along the primary and secondary trabeculae, whereas PHOSPHO1 was restricted to the growth plate chondro‐osseous junction, near sites of early mineral deposition [27, 55].

Consistent with previous studies, we found that the complete deletion of *Alpl* resulted in a failure to form SOCs, with instead large nests of hypertrophic chondrocytes, suggestive of a defect in cartilage matrix production [12, 56]. Such observations have also been reported in patients with severe HPP [12]. Interestingly, this failure has previously been shown to be rescued upon administration of a soluble chimeric alkaline phosphatase [56], and herein we found that in our heterozygous mice, one allele of *Alpl* was sufficient to support SOC formation by 6‐weeks of age. The SOC defect in our *Alpl^Prx1/Prx1^* mice may however, have led to the compensatory formation of growth plate bridges in an attempt to stabilize the epiphysis in these animals. Progressive growth plate narrowing coincides with accrual of perpendicular calcified struts, herein termed growth plate bridges [41]. Indeed, previous digital volume correlation and finite element modelling data support a mechanical role for growth plate bridges by revealing that their clustering is linked to elevated local stress/strain levels, as well as increased stress dissipation distal to the growth plate [41, 57].

Further histological abnormalities were observed in the growth plates of our animals. Previous studies have shown both PHOSPHO1 and ALPL expression in hypertrophic growth plate chondrocytes and at the growth plate – metaphysis interface, where their presence facilitates the mineralization process [30, 58, 59]. PHOSPHO1 ablation significantly alters the growth plate lipidome [32] and results in growth plate abnormalities [11]. We observed distention of the growth plate cartilage into the metaphyseal region of *Alpl^Prx1/Prx1^;Phospho1^−/−^* mice, together with an accumulation of disorganized, SOX9‐positive chondrocytes. Previous work by Lui et al. has shown that the downregulation of SOX9 in hypertrophic chondrocytes is associated with an increase in key osteoblast gene expression, with further lineage tracing experiments in *Sox9* transgenic mice revealing a significant reduction in chondrocyte transdifferentiation into osteoblasts [60]. Our persistent SOX9 expression at 3‐weeks of age with *Alpl* deletion therefore likely reflects a suppression of hypertrophic chondrocyte‐to‐osteoblast transdifferentiation during endochondral ossification which subsequently impairs metaphyseal bone structure [60, 61]. This may be coupled with excessive cartilage and bone resorption, as indicated by our osteoclast data in our 6‐week animals, however these alterations are likely secondary to the impaired matrix mineralization and not an inherent defect in osteoclast differentiation. Impaired cartilage remodeling due to defective VEGF‐mediated vascular invasion could also result in a failure to recruit chondroclasts and osteogenic progenitors to permit matrix turnover [62, 63]. Indeed, inactivation of VEGF and suppressed blood vessel invasion results in hypertrophic zone expansion [64, 65]. Future studies could therefore examine these mechanisms further, including exploring osteoclast recruitment and activity, and any effects on angiogenic‐osteogenic coupling at this interface.

Such abnormalities were observed in 6‐week‐old animals, and particularly with *Phospho1* ablation in *Phospho1* ^−/−^ and *Alpl^wt/Prx1^;Phospho1^−/−^* mice, whereby enlarged populations of disorganized chondrocytes with persistent SOX9 expression in the peripheral regions of the growth plate were observed, particularly in the medial condyle. These resembled that seen in the growth plates of mice with conditional deletion of ciliary Intraflagellar Transport Protein 88 (IFT88), highlighting a role for the cilia in ensuring coordinated growth plate dynamics [66]. Increased physiological loading inhibited peripheral growth plate ossification in WT mice, also demonstrating the sensitivity of growth plate dynamics in response to mechanical loads [66]. Indeed, it is plausible that the differences observed herein in the medial peripheral growth plate may result in defective load transfer from epiphyseal regions, ultimately contributing to the differences observed in tibial shape of *Phospho1* ^−/−^ and *Alpl^wt/Prx1^;Phospho1^−/−^* mice. It is known that loading exerts significant lasting modifications in tibial shape [67] and this may be further compounded on a hypomineralized skeleton that is unable to withstand physiological load, considered a key factor in the bowing observed in *Phospho1* ^−/‒^ mice [36].

Alongside tibial shape changes, increased cortical porosity was also observed in *Phospho1*‐deficient animals. Cortical porosity is a major risk factor for fragility fractures, with previous research highlighting distinct spatial variation in cortical porosity and canal density, regulated by bone‐derived VEGF [43]. This is associated with regional vulnerability, as is observed in the tibiofibular junction of *Phospho1^−/‒^* and *Alpl^wt/Prx1^;Phospho1^−/‒^* mice here. Such vulnerabilities are likely exacerbated by the significant mineralization defects observed in *Phospho1^−/‒^* and *Alpl^wt/Prx1^;Phospho1^−/−^* mice with von Kossa staining and BSSEM, akin to osteomalacia, often characterized by broad bands of osteoid with partial or complete failure of calcification [68]. Interestingly, Raman spectroscopic analysis of the epiphysis revealed sex‐specific alterations in mineral and ECM composition of all genotypes, consistent with previously reported divergences in skeletal ECM organization [49]. Reduced hydroxyapatite crystallinity and increased carbonate content of primary teeth from HPP patients have been reported, which opposes our epiphyseal results observed in our *Alpl^Prx1/Prx1^* mice [69]. However, sex‐specific differences and analysis on *Alpl*‐deficient bone are yet to be investigated. Whilst our findings in *Phospho1^−/−^* females appear consistent with our previous analyses [25, 43], the increased matrix carbonation in male animals is at odds. The involvement of sex hormones in contributing to any sex‐specific differences is plausible, and certainly there are numerous studies detailing the role that these may play in osteoblast function and ECM formation [70, 71, 72, 73, 74]. However, as limited sex differences were observed in other parameters, these findings may reflect our small sample size. Overall, we found that dual deletion of *Alpl* and *Phospho1* resulted in changes in collagen configuration which favoured matrix carbonation and as a result, compromised hydroxyapatite mineralization at both initiation (PHOSPHO1‐dependent) and propagation (TNAP‐dependent) stages, resulting in a structurally weak matrix with reduced mechanical integrity [75]. Future work could examine the role of mechanical loading in these animals to determine the effects of dual TNAP and PHOSPHO1 deletion on bone mechanical properties.

Our lack of significant differences in serum levels of PP_i_ or ALP is at odds with previous studies in which decreased circulating levels of ALP and increased circulating PP_i_ have been observed in mice with a *Prx*‐*Cre* specific deletion of *Alpl* [76, 77]. This is likely attributable to age; however further examination of circulating and tissue mineralization regulators could provide valuable mechanistic insights to further distinguish between defective mineralization and altered remodeling. Such analyses were beyond the scope of this study, largely due to limited available serum volumes from the animals examined.

Our recent work in human primary osteoblasts showed that high levels of extracellular P_i_ (eP_i_) decreased expression of the phosphate transporters *Slc20a1* and *Slc20a2* [78]. Similarly, eP_i_ decreased *Phospho1* and *Alpl* gene expression, whilst elevating *Enpp1* and *Ank*, consistent with the concept that osteoblasts have an eP_i_ feedback mechanism to ensure physiological mineralization [78, 79]. This is broadly consistent with our findings herein in which gene expression of both *Slc20a1* and *Slc20a2* was increased with *Phospho1* deletion. Previous studies have shown that conditional ablation of *Slc20a1* in chondrocytes, in combination with global *Phospho1* deletion, worsened the skeletal phenotype than the individual gene deletions, which was marked by increased osteoid, growth plate disorganization and reduced mechanical properties [9]. Similarly, bone quality and strength was impaired in mice lacking *Slc20a2*, suggesting this as a regulator of mineralization [80], however, the mechanism for P_i_ transport into the MV remains unclear as several proteomic studies have been unable to detect PiT‐1 or PiT‐2 (encoded by *Slc20a1* and *Slc20a2*, respectively) within MV [81]. The upregulation of *Smpd3* and *Chkb* in *Phospho1*‐deficient animals likely reflects a compensatory attempt to increase substrate availability (phosphocholine) for PHOSPHO1‐mediated P_i_ generation, reinforcing the central role of PHOSPHO1 in intravesicular mineral initiation [3, 82, 83, 84].

Interestingly, in the single stillborn global double knockout mouse, mineralization of the axial skeleton was observed and attributed to the actions of ENPP1, the enzyme that breaks down extracellular ATP into AMP and pyrophosphate (PP_i_), conferring a compensatory role in the absence of TNAP [11]. Indeed, dual deletion of *Alpl* and *Enpp1* corrects mineralization defects observed upon single gene deletion and is thought to be due to a normalization of PP_i_ concentrations [85]. More recently, the synergistic function of TNAP and ENPP1 in ATP hydrolysis for a permissive P_i_/PP_i_ ratio has been defined [86]. We found a reduction in ENPP1 expression with *Alpl* deletion at both the gene and protein level, suggesting an attempt to correct the local P_i_ environment. Whilst other mechanisms may contribute to these results, we did not observe any alterations in the expression of several genes associated with the RANK/RANKL/OPG or WNT signaling pathways. Additional mechanistic studies incorporating more comprehensive transcriptomic and proteomic analyses could identify further pathways responsible for the observed mineralization phenotypes.

In summary, our data confirm the essential roles of TNAP and PHOSPHO1 for effective biomineralization and uncover their spatially distinct and fundamental functions in long bone development. Our in vivo evidence supports a spatially and mechanistically distinct model of TNAP and PHOSPHO1 function whereby each phosphatase preferentially affects different anatomical structures. PHOSPHO1 acts within MVs to initiate mineral deposition through localized P_i_ generation, while TNAP functions extracellularly to hydrolyze PP_i_ and permit propagation of hydroxyapatite crystals. Loss of PHOSPHO1 primarily impairs mineral initiation and compromises growth plate formation leading to marked cortical bone abnormalities in the diaphysis. Conversely, TNAP disrupts extracellular mineral expansion, particularly in regions with increased bone turnover, thus preferentially results in a compromised SOC, epiphysis and metaphysis. Simultaneous ablation therefore results in the severe phenotype observed in the double mutants. By identifying synergistic phosphatase roles, this research informs enzyme replacement and gene therapy strategies, with potential to reduce healthcare costs and improve quality of life globally.

## Author Contributions

LEB, KAS, CF, JLM, LS, SN, BLF designed research; LEB, AS, SD, JK, SNJ, NC, LAEE, MK, WP, CEC performed research; LEB, AS, SD, JK analyzed data; LEB, KAS wrote the manuscript. All authors reviewed and edited the manuscript.

## Funding

The authors would like to acknowledge the UKRI Medical Research Council (MRC) for funding to KAS (MR/V033506/1 & MR/R022240/2), the Biotechnology and Biological Sciences Research Council (BBSRC) for Institute Strategic Programme Grant Funding to CF/SNJ (BBS/E/D/10002071 & BBS/E/RL/230001C) and for supporting LAS via a Discovery Fellowship (BB/X009904/1), and NIDCR (R01DE032334) for funding BLF.

## Ethics Statement

All tissue isolation and experimental procedures were performed in accordance with the UK Animals (Scientific Procedures) Act of 1986 and regulations set by the UK Home Office and local institutional guidelines at the University of Brighton.

## Conflicts of Interest

The authors declare no conflicts of interest.

## Supporting information




**Supporting File**: advs77671‐sup‐0001‐SuppMat.docx.

## Data Availability

The data that support the findings of this study are available from the corresponding author upon reasonable request.
