# Supplementary material for: TNAP and PHOSPHO1 Function Synergistically to Afford Critical Control Over the Mineralization of the Postnatal Murine Skeleton
Source: Adv Sci (Weinh). 2026 Sep 9:e77671. Online ahead of print. doi: 10.1002/advs.77671 (PMC13558897; doi:10.1002/advs.77671)
Supplement: Supplementary file 1 [file ADVS-9999-e77671-s001.docx]

**Supplementary Figure 1. Confirmation of knockout in femoral samples.** Western blot on protein lysates of wild-type (WT), *Alpl^Prx1/Prx1^*, *Phospho1^-/-^*, *Alpl^wt/Prx1^;Phospho1^-/-^*, and *Alpl^Prx1/Prx1^;Phospho1^-/-^* mice shows the loss of PHOSPHO1 expression in *Phospho1^-/-^* containing genotypes and loss of TNAP expression in *Alpl^Prx1/Prx1^* containing genotypes, normalised to β-actin. *Alpl^wt/Prx1^;Phospho1^-/-^* shows a reduction in TNAP expression compared to WT and *Phospho1^-/-^*.

**Supplementary Figure 2. Whole mount imaging of entire skeletons at PN1 and 3-weeks-old, and histological analysis of 3-week-old mice.** Representative images of wild-type (WT), *Alpl^Prx1/Prx1^*, *Phospho1^-/-^*, *Alpl^wt/Prx1^;Phospho1^-/-^*, and *Alpl^Prx1/Prx1^;Phospho1^-/-^* mice at (**A**) postnatal day 1 and (**B**) 3 weeks old. Arrows indicate mineralisation in all genotypes except for *Alpl^Prx1/Prx1^;Phospho1^-/-^* double knockout mice. Scale bar = 1cm. (**C**) Representative images of Haematoxylin/Eosin and (**D**) Toluidine Blue staining of femurs from each genotype at 3 weeks of age. Arrows indicate enlarged growth plate extending into the metaphyseal region. Scale bar = 200 µm. GP = growth plate, SOC = secondary ossification centres, TB = metaphyseal trabecular bone.

**Supplementary Figure 3. No difference in gross morphology or serum biochemistry of 6-week-old animals.** (**A**) Representative images of wild-type (WT), *Alpl^Prx1/Prx1^*, *Phospho1^-/-^*, *Alpl^wt/Prx1^;Phospho1^-/-^*, and *Alpl^Prx1/Prx1^;Phospho1^-/-^* mice. Scale bar = 2cm. (**B)** Serum biochemistry analysis for **(i & ii**) ELISA detecting alkaline phosphatase (ALP) and (**iii & iv**) colorimetric assay detecting pyrophosphate (PP_i_) in males and females, respectively. Data are presented as mean ± SEM with points showing individual animals (n=3-7/genotype/sex).

**Supplementary Figure 4. Additional trabecular parameters from the epiphyseal region of 6-week-old male and female mice.** μCT analysis of trabecular parameters from wild-type (WT), *Alpl^Prx1/Prx1^*, *Phospho1^-/-^*, *Alpl^wt/Prx1^;Phospho1^-/-^*, and *Alpl^Prx1/Prx1^;Phospho1^-/-^* mice in (**A**) males and (**B**) females, including (**i**) bone surface, (**ii**) bone surface to bone volume fraction (BS/BV), (**iii**) trabecular thickness (Tb.Th), (**iv**) trabecular spacing (Tb.Sp), (**v**) connectivity density (Conn. Dn), and (**vi**) fractal dimension. Data are presented as mean ± SEM with points showing individual animals (n=5-7/genotype/sex). *= *p* < 0.05, **= *p* < 0.01, ***= *p* < 0.001, ****= *p* < 0.0001.

**Supplementary Figure 5. Raman spectra for 6-week-old male mice.** Raman spectra collected within the fingerprint region from 700 cm-1 to 1750 cm-1 were obtained from the tibial epiphyses of (**A**) wild-type (WT), (**B**) *Alpl^Prx1/Prx1^,* (**C**) *Phospho1^-/-^*, and (**D**) *Alp^wt/Prx1^;Phospho1^-/-^* male mice (n=3/sex/genotype). Presented as vector normalised class means spectra (n=75 individual spectra) ±SD. Second derivative calculation (**E**) facilitated the identification of Raman bands corresponding to extracellular matrix and mineral components, shown in zoomed second derivative spectra (**F**) which were analysed by spectral deconvolution.

**Supplementary Figure 6. Raman spectra for 6-week-old female mice.** Raman spectra collected within the fingerprint region from 700 cm-1 to 1750 cm-1 were obtained from the tibial epiphyses of (**A**) wild-type (WT), (**B**) *Alpl^Prx1/Prx1^,* (**C**) *Phospho1^-/-^*, and (**D**) *Alp^wt/Prx1^;Phospho1^-/-^* female mice (n=3/sex/genotype). Presented as vector normalised class means spectra (n=75 individual spectra) ±SD. Second derivative calculation (**E**) facilitated the identification of Raman bands corresponding to extracellular matrix and mineral components, shown in zoomed second derivative spectra (**F**) which were analysed by spectral deconvolution.

**Supplementary Figure 7. Additional trabecular parameters from the metaphyseal region of 6-week-old mice.** μCT analysis of trabecular parameters from wild-type (WT), *Alpl^Prx1/Prx1^*, *Phospho1^-/-^*, *Alpl^wt/Prx1^;Phospho1^-/-^*, and *Alpl^Prx1/Prx1^;Phospho1^-/-^* mice in (**A**) males and (**B**) females, including (**i**) bone surface, (**ii**) bone surface to bone volume fraction (BS/BV), (**iii**) trabecular thickness (Tb.Th), (**iv**) trabecular spacing (Tb.Sp), (**v**) connectivity density (Conn. Dn), and (**vi**) fractal dimension. Data are presented as mean ± SEM with points showing individual animals (n=5-7/genotype/sex). *= *p* < 0.05, **= *p* < 0.01, ***= *p* < 0.001, ****= *p* < 0.0001.

**Supplementary Figure 8. Additional cortical parameters in 6-week-old mice.** Measurement and statistical analysis heat map in (**A**) male and (**B**) female mice for (**i**) tissue perimeter (T.Pm), (**ii**) bone perimeter (B.Pm), (**iii**) maximum second moments of inertia (*I_max_*) and, (**iv**) minimum second moments of inertia (*I_min_*). Line graphs represent mean ± SEM for wild-type (WT; black), *Alpl^Prx1/Prx1^* (blue), *Phospho1^-/-^* (green) and *Alpl^wt/Prx1^;Phospho1^-/-^* (orange) mice (n=5-7/genotype/sex). Graphical heat map summarises statistical differences at specific matched locations along the tibial length (15–90%). Red = *p* < 0.001, green = *p* < 0.01, yellow = *p* < 0.05, grey = *p* > 0.05 (not significant).

**Supplementary Figure 9. Additional genes analysed by RT-qPCR & BSEM images.** (**A**) RT-qPCR of additional genes from the femurs of wild-type (WT), *Alpl^Prx1/Prx1^*, *Phospho1^-/-^*, *Alpl^wt/Prx1^;Phospho1^-/-^*, and *Alpl^Prx1/Prx1^;Phospho1^-/-^* mice, in males and females. Data are presented as mean ± SEM with points showing individual animals (n=5-7/genotype/sex). *= *p* < 0.05, **= *p* < 0.01. (**B**) Back scattered scanning electron microscopy images of the cortices. Arrowheads indicate failure of mineralisation foci to propagate within areas of hypomineralisation. Scale bar = 50 µm.

**Supplementary Table 1**: Gross morphological measurements for 6-week-old male and female mice. Data represented as mean ± SEM for *n*=5-7/genotype/sex. * = *p* < 0.05, ** = *p* < 0.01, *** = *p* < 0.001, **** = *p* < 0.0001.

| **Male** | | | | | |
| --- | --- | --- | --- | --- | --- |
|  | **WT** | ***Alpl^Prx1/Prx1^*** | | ***Phospho1^-/-^*** | ***Alpl^wt/Prx1^;***  ***Phospho1^-/-^*** |
| **Weight (g)** | 24.39  ±0.78 | 23.15  ±0.66 | | 21.11  ±0.51 | 22.07  ±0.60 |
| Multiple comparisons test results (Tukey’s HSD) | | | | | |
| WT vs. *Alpl^Prx1/Prx1^*  WT vs. *Phospho1*^-/-^  WT vs. *Alpl^wt/Prx1^;Phospho1^-/-^*  *Alpl^Prx1/Prx1^* vs. *Phospho1*^-/-^  *Alpl^Prx1/Prx1^* vs. *Alpl^wt/Prx1^;Phospho1^-/-^*  *Phospho1*^-/-^ vs. *Alpl^wt/Prx1^;Phospho1^-/-^* | | | ns  **  ns  ns  ns  ns | | |
|  | **WT** | ***Alpl^Prx1/Prx1^*** | | ***Phospho1^-/-^*** | ***Alpl^wt/Prx1^;***  ***Phospho1^-/-^*** |
| **Nose-to-tail length (cm)** | 16.48  ±0.16 | 16.80  ±0.16 | | 15.64  ±0.13 | 15.54  ±0.244 |
| Multiple comparisons test results (Tukey’s HSD) | | | | | |
| WT vs. *Alpl^Prx1/Prx1^*  WT vs. *Phospho1*^-/-^  WT vs. *Alpl^wt/Prx1^;Phospho1^-/-^*  *Alpl^Prx1/Prx1^* vs. *Phospho1*^-/-^  *Alpl^Prx1/Prx1^* vs. *Alpl^wt/Prx1^;Phospho1^-/-^*  *Phospho1*^-/-^ vs. *Alpl^wt/Prx1^;Phospho1^-/-^* | | | ns  **  **  ***  ***  ns | | |
|  | **WT** | ***Alpl^Prx1/Prx1^*** | | ***Phospho1^-/-^*** | ***Alpl^wt/Prx1^;***  ***Phospho1^-/-^*** |
| **Bone length (mm)** | 16.82  ±0.10 | 16.19  ±0.10 | | 14.50  ±0.31 | 13.32  ±0.32 |
| Multiple comparisons test results (Tukey’s HSD) | | | | | |
| WT vs. *Alpl^Prx1/Prx1^*  WT vs. *Phospho1*^-/-^  WT vs. *Alpl^wt/Prx1^;Phospho1^-/-^*  *Alpl^Prx1/Prx1^* vs. *Phospho1*^-/-^  *Alpl^Prx1/Prx1^* vs. *Alpl^wt/Prx1^;Phospho1^-/-^*  *Phospho1*^-/-^ vs. *Alpl^wt/Prx1^;Phospho1^-/-^* | | | ns  ****  ****  ****  ****  * | | |
| **Female** | | | | | |
|  | **WT** | ***Alpl^Prx1/Prx1^*** | | ***Phospho1^-/-^*** | ***Alpl^wt/Prx1^;***  ***Phospho1^-/-^*** |
| **Weight (g)** | 19.51  ±0.37 | 17.86  ±0.82 | | 17.22  ±0.53 | 18.13  ±0.46 |
| Multiple comparisons test results (Tukey’s HSD) | | | | | |
| WT vs. *Alpl^Prx1/Prx1^*  WT vs. *Phospho1*^-/-^  WT vs. *Alpl^wt/Prx1^;Phospho1^-/-^*  *Alpl^Prx1/Prx1^* vs. *Phospho1*^-/-^  *Alpl^Prx1/Prx1^* vs. *Alpl^wt/Prx1^;Phospho1^-/-^*  *Phospho1*^-/-^ vs. *Alpl^wt/Prx1^;Phospho1^-/-^* | | | ns  *  ns  ns  ns  ns | | |
|  | **WT** | ***Alpl^Prx1/Prx1^*** | | ***Phospho1^-/-^*** | ***Alpl^wt/Prx1^;***  ***Phospho1^-/-^*** |
| **Nose-to-tail length (cm)** | 16.05  ±0.12 | 15.96  ±0.22 | | 15.07  ±0.22 | 15.57  ±0.13 |
| Multiple comparisons test results (Tukey’s HSD) | | | | | |
| WT vs. *Alpl^Prx1/Prx1^*  WT vs. *Phospho1*^-/-^  WT vs. *Alpl^wt/Prx1^;Phospho1^-/-^*  *Alpl^Prx1/Prx1^* vs. *Phospho1*^-/-^  *Alpl^Prx1/Prx1^* vs. *Alpl^wt/Prx1^;Phospho1^-/-^*  *Phospho1*^-/-^ vs. *Alpl^wt/Prx1^;Phospho1^-/-^* | | | ns  **  ns  *  ns  ns | | |
|  | **WT** | ***Alpl^Prx1/Prx1^*** | | ***Phospho1^-/-^*** | ***Alpl^wt/Prx1^;***  ***Phospho1^-/-^*** |
| **Bone length (mm)** | 16.46  ±0.09 | 15.68  ±0.13 | | 14.21  ±0.15 | 13.18  ±0.28 |
| Multiple comparisons test results (Tukey’s HSD) | | | | | |
| WT vs. *Alpl^Prx1/Prx1^*  WT vs. *Phospho1*^-/-^  WT vs. *Alpl^wt/Prx1^;Phospho1^-/-^*  *Alpl^Prx1/Prx1^* vs. *Phospho1*^-/-^  *Alpl^Prx1/Prx1^* vs. *Alpl^wt/Prx1^;Phospho1^-/-^*  *Phospho1*^-/-^ vs. *Alpl^wt/Prx1^;Phospho1^-/-^* | | | *  ****  ****  ****  ****  ** | | |

**Supplementary Table 2**: Western blot and immunohistochemistry (IHC) antibodies and details

| **Antibody** | **Source** | **Concentration** | **Company & catalogue number** | **Application** |
| --- | --- | --- | --- | --- |
| β-actin (Conjugated with HRP) | Rabbit | 1:1000 | Cell Signaling Technology | Western blot |
| MMP13 | Rabbit | 1:200 | Abcam (ab39012) | IHC |
| PHOSPHO1 | Human | 1:250 | Bio-Rad (HCA093) | Western blot |
| SOX9 | Rabbit | 1:500 | Merck (AB5535) | IHC |
| OPN | Rabbit | 1:200 | Kerafast (LF-175) | IHC |
| ENPP1 | Goat | 1:200 | Abcam (ab40003) | IHC |
| TNAP | Human | 1:500 | R&D Systems (MAB1448) | Western blot |
| anti-Human IgG (H/L): HRP | Goat | 1:1000 | Bio-Rad (5172-2504) | Western blot (secondary) |

**Supplementary Table 3.** Primer sequences used for RT-qPCR

| **Gene** | **Forward sequence** | **Reverse sequence** |
| --- | --- | --- |
| ***Alpl*** | GGGACGAATCTCAGGGTACA | AGTAACTGGGGTCTCTCTCTTT |
| ***Ank*** | TCGCTGCCTTCCCTTTTATG | GGTGACTGTGAAGCAAAATGG |
| ***Atpb5*** | ATGCCACTTCCAAGGTAGCG | GCAACAGTCAGACCAGTCAGA |
| ***βactin*** | GATGTGGCACCACACCTTCT | GGGGTGTTGAAGGTCTCAAA |
| ***Bglap*** | CCGGGAGCAGTGTGAGCTTA | TAGATGCGTTTGTAGGCGGTC |
| ***Chkb*** | GCAAGACCCACGGACTACC | CAGGGAGTACCGACTGATCTC |
| ***Enpp1*** | GCTAATCATCAGGAGGTCAAG | CTGGTAGAATCCCGTCAATC |
| ***Enpp6*** | GTAGTCATCTTGGACCCTCTCATACTG | GTGTGAGCTCTTACATGTGGACAGA |
| ***Lrp5*** | CCTCACCATTGATTATGCCGACC | GATCGTCAGCTATCACCATGCG |
| ***Lrp6*** | CTGAATGCTGACAACAGGACCTG | GACGTTCCGAAGGCTGTGGATA |
| ***Opg*** | AAATTGGCTGAGTGTTTTGGTG | CTGTGTCTCCGTTTTATCCTCTC |
| ***Phex*** | CTAACCACCACTCCCACTT | CCAATAGACTCCAACCTGAAGA |
| ***Phospho1*** | GCCTGCGTGCCACCTAT | CCTGCTCACCCAGGTACTTAAA |
| ***Rank*** | GGGTGATTTTCTTTTGGTGGGTC | TGCTCGTGATAACTATGCCTGTG |
| ***Rankl*** | AAATTGGCTGAGTGTTTTGGTG | CTGTGTCTCCGTTTTATCCTCTC |
| ***Slc20a1*** | GGCTCAGGTGTAGTGACCCT | CACATCTATCAAGCCGTTCC |
| ***Slc20a2*** | CCATCGGCTTCTCACTCGT | AAACCAGGAGGCGACAATCT |
| ***Smpd3*** | ACACGACCCCCTTTCCTAATA | GGCGCTTCTCATAGGTGGTG |
| ***Sost*** | TGAGAACAACCAGACCATGAAC | TCAGGAAGCGGGTGTAGTG |
| ***Spp1*** | GAGAGCCAGGAGAGTGCCGA | GCTTTGGAACTTGCTTGACTATCG |
